# Consideration of a new approach to clarify the mechanism formation of AgNPs, AgNCl and AgNPs@AgNCl synthesized by biological method

**DOI:** 10.1186/s11671-023-03777-w

**Published:** 2023-02-01

**Authors:** Railean Viorica, Pomastowski Pawel, Tomasz Płociński, Michał Gloc, Renata Dobrucka, Krzysztof Jan Kurzydłowski, Buszewski Boguslaw

**Affiliations:** 1 Department of Infectious, Invasive Diseases and Veterinary Administration, Institute of Veterinary Medicine, Nicolaus Copernicus University in Torun, Gagarina 7, 87-100 Toruń, Poland; 2 Centre for Modern Interdisciplinary Technologies, Nicolaus Copernicus University in Torun, Wileńska 4, 87-100 Toruń, Poland; 3 Faculty of Materials Science and Engineering Warsaw, University of Technology, Ul. Wołoska 141, 02-507 Warsaw, Poland; 4 Department of Non-Food Products Quality and Packaging Development, Institute of Quality Science, Poznań University of Economics and Business, Al. Niepodległości 10, 61-875 Poznan, Poland; 5 Faculty of Mechanical Engineering, Bialystok University of Technology, Ul. Wiejska 45C, 15-351 Białystok, Poland; 6 Department of Environmental Chemistry and Bioanalytics, Faculty of Chemistry, Nicolaus Copernicus University in Torun, Gagarina 7, 87-100 Toruń, Poland

**Keywords:** *Lactobacillus* isolates, Silver chloride nanoparticles, Hybrid, Organic coats, Mechanism formation, Biosynthesis

## Abstract

The biological methods are considered as environmental-eco-friendly methods for the silver nanocomposites mediation and are widely used in this context. However, the biological methods go along with the relevant limitations, for instance simultaneous synthesis of silver chlorides (AgNCl) type during the AgNPs mediation process. Therefore, the present research is coming to summarize several aspects in this context. Firstly, to present the possible promotion of the sustainable development using bioactive source (e.g. milk) as a source of two different available and new *lactobacillus* strains (*Lactobacillus curvatus* and *Lactobacillus fermentum)*. Secondly, to show the ability of the respective isolates to be involved in mediation of various biosilver nanocomposites ((Bio)NCs) synthesis. Moreover, at this stage, for the first time, two (Bio)NCs mediation methods, called “direct method” and “modified method”, have been developed, thus three types (AgNPs, AgNCl and AgNP@AgNCl) of nanocomposites mediated by two different *Lactobacillus* isolates take place. The interdisciplinary approach included using several spectroscopic, microscopic, spectrometric and thermogravimetric methods demonstrated that all six synthesized nanoparticles (three AgNPs, AgNCl and AgNP@AgNCl types from each source) consist of complex structure including both metallic silver core as well as organic surface deposits. The spectrometric technique allowed to identification of the organics branching surface, naturally secreted by the used *Lactobacillus* isolates during the inoculation step, suggesting the presence of amino-acids sequences which are direct connected with the reduction of silver ion to metal silver, and subsequently with the formation of coated (Bio)NCs and nucleation process. Moreover, based on the obtained results, the mediation mechanism of each (Bio)NCs has been proposed, suggesting that the formation of AgNPs, AgNCl and AgNP@AgNCl types occurs in different manners with faster synthesis firstly of AgNCl, then of the AgNPs type. No differences between the (Bio)NCs synthesized by two different *Lactobacillus* isolates have been noticed indicating no discrepancies between metabolites secreted by the respective sources.

**Graphical abstract:**

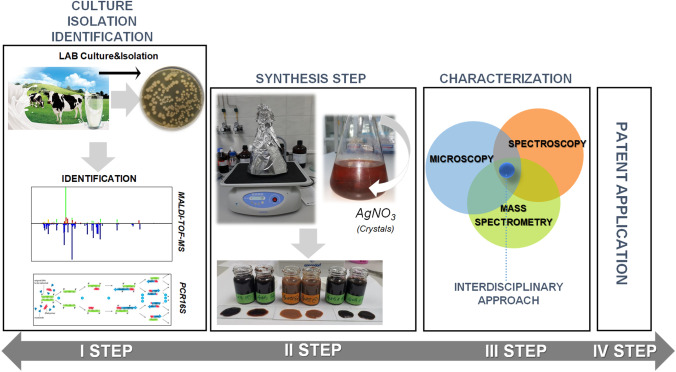

**Supplementary Information:**

The online version contains supplementary material available at 10.1186/s11671-023-03777-w.

## Introduction

In recent years, nanotechnology has become a part of almost every field of science (e. g. physics chemistry, biology, engineering etc.) allowing us to live a better and easier life. Nanotechnology is required for the measuring, monitoring, assembling and mediating at Nano scale by involving interdisciplinary approach for the structure, composition and stability characterization as well as medical area application [1]. On the other hand, sustainable development is attracting more attention and certainly, the pursuit of the new opportunities to involve the resulting products (by-products) in the recycling process has developed high interest. Once the ecological problems are in continuously increasing, giving a new life application of the biological source (e.g. milk) has become a requirement. In fact, both milk as well as whey are a source of healthy nutrients and microorganisms aimed to contribute directly or indirectly to the human life.

In addition to that, many study including our works have been demonstrated firstly, the possibility to use the available whey as a cheap by-product source for the isolation of the safe lactic acids bacterial strains (LAB) and secondly, the involving of the respective strains in the nanocomposites (NCs) mediations (AgNPs, ZnONPs, etc.). [2, 3] Since this aspect is crucial when associated with the emergence of environmental problems, it is essential to point out the indispensability of using of biological methods for the NCs formation. In the same order of ideas, a fundamental aspect on using biological methods for the NCs mediation via involving bio-source (bacteria, fungi, plants etc.) is an integrated and adequate characterization of the (Bio)silver products. Certainly, the biological methods are accompanied also by the limitations regarding the reproducibility process; for instance, during the AgNPs synthesis the formation of AgNCl takes place simultaneously [4–6]. In view of these statements, the interpretation of the results concerning the physico-chemical characterization, should be made with caution, since AgNPs@AgNCl might be produced as a hybrid form. Moreover, the determination of organic deposit naturally covering the metallic silver core and impact of the respective branching coats on the stability of the NCs are poorly discussed.

Therefore, the present study has proposed to attend several goals; (1) firstly to promote the recycling approach by using LAB isolates from the milk as a bioactive source for the synthesis of several (Bio)nanocomposites; (2) secondly, to mediate silver nanocomposites and develop the method for separation of AgNPs and AgNCl nanocomposites; (3) thirdly, to employ an adequate characterization based on interdisciplinary approach using complimentary methods such as spectroscopy, microscopy, spectrometry and thermal analysis in order to clarify the mechanism formation of biologically synthesized silver nanoparticles, silver chloride nanoparticles and their hybrids; (4) finally and particularly, the present study is coming to indeed shed further light onto innovative approach of (Bio) silver-nanocomposites concerning structure, organic coats onto/into silver core, stability of the biocolloids in time and discrepancies between LAB strains as well as NCs types (AgNPs, AgNCl and AgNP@AgNCl). In another train of thoughts, ones nowadays increasing ecological problems have gained interest worldwide, the detailed information obtained based on the complex interdisciplinary analysis gives a new scientific approach concerning the biological synthesis of silver nanomaterial naturally coated by organic core.

## Material and methods

### Identification of lactic acid bacterial (LAB) isolates—used for the further synthesis as a safe source

The isolation and identification of LAB sources (*Latilactobacillus curvatus* MEVP1 [OM736187] and *Limosilactobacillus fermentum* MEVP2[OM736188*]*) have been performed following the protocols using the De Man, Rogosa and Sharpe agar medium (MRSA) as was described by Railean et al. [7]. The strains’s isolation was carried out based on serial dilutions methods while identification—on the classical “dry droplet” method, in compliance with fulfilled modifications. The smeared bacterial pellets have been overlaid by the HCCA matrix onto a polished steel target plate in triplicates and analyzed using the MALDI BioTyper database (Bruker Daltonik). The recording of the data has been accomplished in the molecular weight range by summing up the shot stages with a minimum laser power of 40% and the acceleration voltage in MALDI MS was 25 kV; the results values presented as a “logRQ”—indicate the degree of similarity between profiles of our interest and database provided by the manufacturer (score > 1.7—positive results whereas < 1.7—negative results). The calibration of the system was acquired with the bacteria calibration standard (Bruker Bacterial Test Standard) according to guidelines presented by the manufacturer and main spectra (MSP) has been recorded by MALDI BioTyper Compass Explorer software as was described by Pomastowski et al. [8]. The dendrogram similarity has been generated by the BioTyper MSP Dendrogram Creation Standard Method that includes the processing/comparison of the obtained MSP to MSP library; the relationship of the microorganisms was respected with a maximum value 1.000 as was suggested by the manufacturer protocol. Then, the *Lactobacillus* isolates have been deposited in the Polish Collection of Microorganisms (PCM).

### Silver nanocomposites (AgNPs, AgNCl, AgNPs@AgNCl) types mediation

The *Latilactobacillus curvatus* MEVP1 [OM736187] (1C) and *Limosilactobacillus fermentum* MEVP2 [OM736188]) (4A) isolates have been used for the synthesis of six different nanocomposites. Using both bacterial strains it has been synthesized into three types of nanoparticles of each: AgNPs, AgNCl, AgNPs H (AgNPs@AgNCl—hybrid). All six synthesized nanocomposites (AgNPs 1C, AgNPs 4A, AgNCl 1C, AgNCl 4A, AgNPs H 1C, AgNPs H 4A) were named based on the bacteria abbreviation presented in parentheses above: (1C) and (4A). For this purpose, the protocol previously described by our group has been followed, with the suitable modifications [7].

**Fig. 1:**
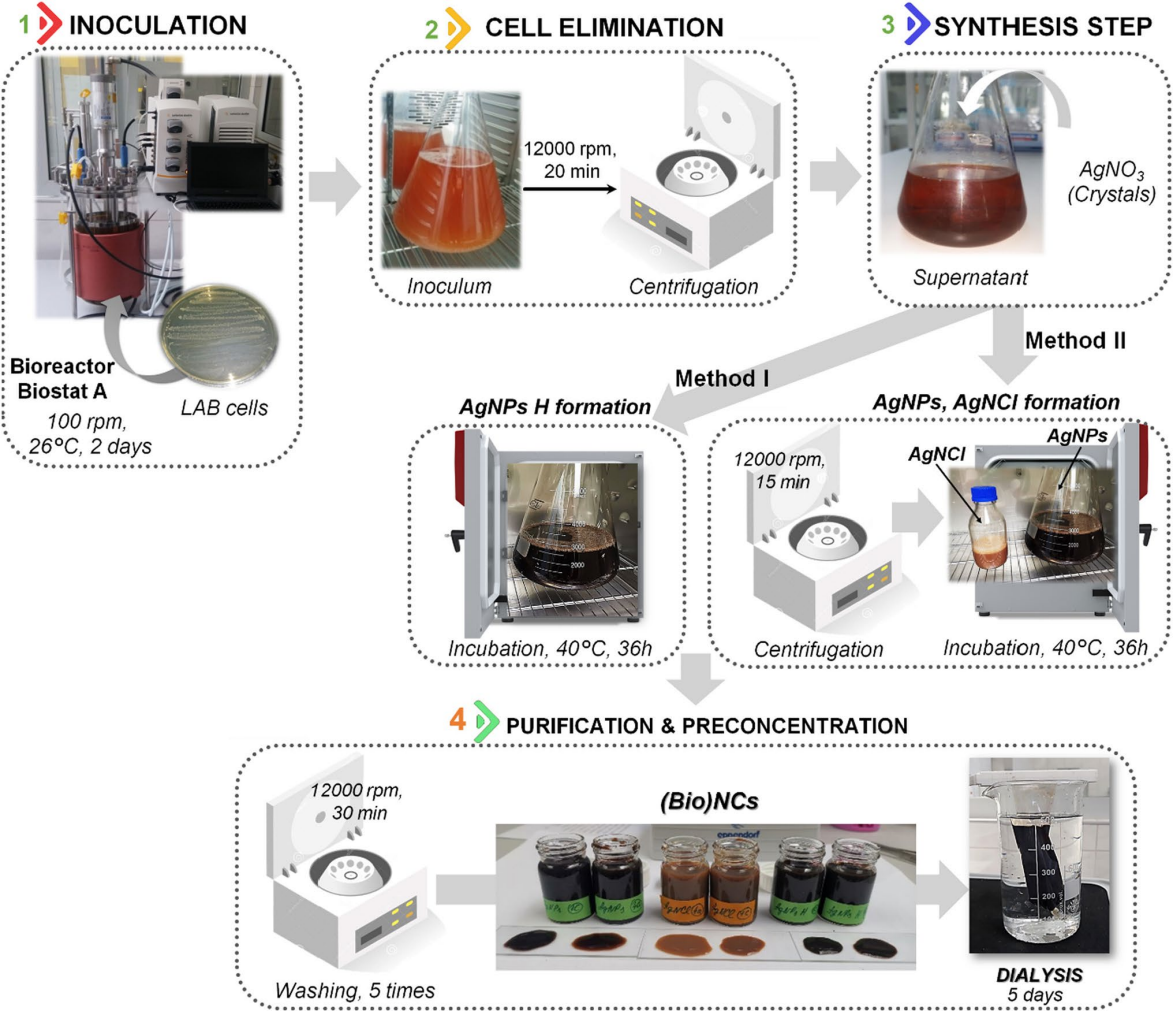
Schematic illustration showing the involved steps for the (Bio)NCs types mediations including both “direct method” (Method I) as well as “modified method” (Method II)

In the current research 5 mM of AgNO_3_ was used as a final concentration and two methods have been performed to obtain 3 types of nanocomposites: *method I* called the “direct method” (synthesis of the hybrid nanocomposites—AgNPs@AgNCl (AgNPs H)) and *method II*—“modified method”, that includes separation of the AgNPs and AgNCl nanocomposites. A scheme of synthesis protocol is presented in Fig. 1.

The synthesis protocol involved 4 steps for both “direct method” as well as for the “modified method”. Since the manufacturer recommends De Man, Rogosa and Sharpe medium (MRS) as an optimal growth medium which stimulates the luxuriant growth of Lactobacilli, in case of both methods the respective medium has been chosen. I STEP includes cultivation of Lactobacilli strains; the bacterial strains were inoculated at 26 °C for 48 h (100 rpm) using bioreactor Biostat A (Sartorius Stedim Biotech, Germany) in order to receive rich amount of metabolites that will be naturally involved in the synthesis of nanocomposites. Then, during the II STEP, the bacterial cells have been eliminated by centrifugation method (10,000 rpm for 15 min) and during the next stage, (III STEP) rich post-culture medium has been combined with AgNO_3(s)_ (5 mM final concentration). In case of *“direct method”*, the obtained mixture has been placed for additional incubation at 40 °C for 36 h while in case of *“modified method”*—directly after well mixing (1 min) the mixture has been centrifuged (12,000 rpm, 15 min); at this stage the AgNPs (supernatant) and AgNCl (precipitate) were separated from each other. The obtained supernatant solution and precipitate have been incubated (40 °C) for another 36 h in the dark in order to allow the complete synthesis of nanocomposites. At IV STEP, after the achievement of the required time, all nanocomposites obtained by I method (AgNPs@AgNCl) and II method (AgNPs and AgNCl) were concentrated (12,000 rpm for 30 min) and purified with water five times by centrifugation at 12,000 rpm for 30 min. Besides purification, a five day period of dialysis (3 kDa cut-off, Spectrumlab, USA) was conducted in order to eliminate silver ions and low molecular weight metabolites that remained uninvolved in the silver nanocomposites formation.

## Bio(silver) nanocomposites types characterization

For the characterization and differentiation of all the synthesized nanocomposites types concerning elemental composition, size distribution, crystalline nature as well as organic coats onto/into silver core and stability of the biocolloids several methods have been employed.

### Microscopy approach

For this purpose several techniques have been involved: scanning electron microscopy (SEM, LEO 1430 VP, Leo Electron Microscopy Ltd, Cambridge, United Kingdom) coupled with an energy dispersive X-ray (EDX) detector (XFlash 4010, Bruker AXS, Bremen, Germany) for the elemental analysis while transmission electron microscopy (TEM, FEI Tecnai F20 X-Twintool, FEI Europe, Frankfurt/Main, Germany) for metallic size core, crystallinity and nanoparticles morphology. TEM micrographs, high resolution (HRTEM) mode, fast-Fourier-transform (FFT), lattice fringes and diffraction ring patterns (SAED) were determined by the TEM Imaging & Analysis software. The d-spacing has been acquired based on HRTEM images whereas particles size distribution – multiple TEM micrographs and processed via JmageJ software. The histogram size distribution has been performed by ORIGIN Pro/2016 software. Surface morphology imaging of nanocomposites has been recorded via atomic force microscopy (AFM), Veeco SPM (Digital Instrument) equipped with NanoScope IIIa controller and Quadrex, MultiMode microscope, E type scanner with maximum scanning area 10 × 10 × 2.5 µm. The samples have been imaged in tapping mode using HN-NC cantilevers with a resonance frequency of 120 kHz; the scan speed was set to 1.97 Hz. The obtained data have been operated via NanoScope Analysis software V1.4 and the depth histogram row data (size distribution) were derived automatically by the instrument-equipped software and processed by ORIGIN Pro/2016 software.

### Spectroscopy approach

The XRD patterns of the mediated types were recorded and distinguished using a crucial technique in the correct identification of AgNPs and AgNCl – the Powder X-ray diffraction (XRD) technique. The crystalline nature, *d*-spacing have also been determined respecting the Bragg reflection that corresponds to the lattice place characteristic for AgNPs and AgNCl. The obtained data were addressed to the International Crystal Structure Database (ICSD) XRD standards, considered as being the etalon. Additional processing of data was accomplished using ORIGIN Pro/2016 software.

The possible functional groups contained in the organic surface composition naturally coating in/onto metallic silver core were recorded by the FTIR and RAMAN analysis. The FTIR spectra have been recorded via Spectrum 2000 (Perkin-Elmer, Waltham, MA, USA) technique by the KBr disc method while RAMAN - Raman spectrometer (Senterra, Bruker Optik) by dropping method on ZnSe lens (Focal length 50.8 mm, 12 mm, 2.4 mm, IUVO-LASER, Poland). All spectra in case of both FTIR and RAMAN measurements have been registered in the *υ* = 400–4000 cm^−1^ range and processed using ORIGIN Pro/2016 software.

The Zetasizer Nano Series (Malvern Instruments, Great Britain) was used for determination of the dispersion stability and size distribution of biocolloids based on the dynamic light scattering methods. The analysis has been carried out at pH = 7 using water as a solvent; the YV Grade cuvettes served for the hydrodynamic size distribution and Folded Capillary cells for the zeta potential measurements. Before analysis all biocolloids were sonicated (30 min) and measurements were made within 7 days with an interval after day 1, day 2, day 3, day 7; the results are presented in terms of three replicates and expressed by standard deviation (SD ±).

The relative fluorescence quantum yields of synthesized (Bio)nanocomposites ((Bio)NCs) was performed by spectrofluorometer Jasco FP-XX (Japan) and was measured in a commonly used 10 mm pathlength quartz cuvette and recorded in the range of wavelength 250–585 nm (Ex) and 260–600 nm (Em). Contour view and fluorescence emission spectra have been recorded and processed via instrument-equipped software – Interval Data Analysis. Once the samples have been dissolved in water, the water has served as a black.

### Thermogravimetric analysis

The TA Instruments type SDT2960 (Artisan Technology, Champaign, IL, USA) was engaged to investigate the thermal stability of all synthesized (Bio)NCs in the temperature range from 20to 700 °C in nitrogen atmosphere with a heating rate of 10 C/min. For the processing of the data and designing of the TG&DTG curve the TGA-DTA thermal analysis software (version V5 7.0, TA Instruments, New Castle, DE, USA) was employed.

### Spectrometry approach: matrix-assisted laser desorption ionization – time of flight mass spectrometry (MALDI-TOF/TOF–MS)

The MALDI-TOF/TOF-MS equipment was involved in the identification of organic coats (amino acids, peptides sequences) and isotopic distribution ability of the mediated (Bio)NCs according to the method described previously by Railean et al. [7]. For the acquisitions, processing and visualizations of data the instrument-equipped software FlexControl and FlexAnalysis were employed. STATISTICA Software (StatSoft, Krakow, Poland) was used in order to design the tree dendrogram which illustrates hierarchical clustering and correlation between the mediated (Bio)NCs based on the one- and two-dimensional mass spectrometry (MS) profiles.

## Results and discussions

### Identification of *Lactobacilli* strains

**Fig. 2:**
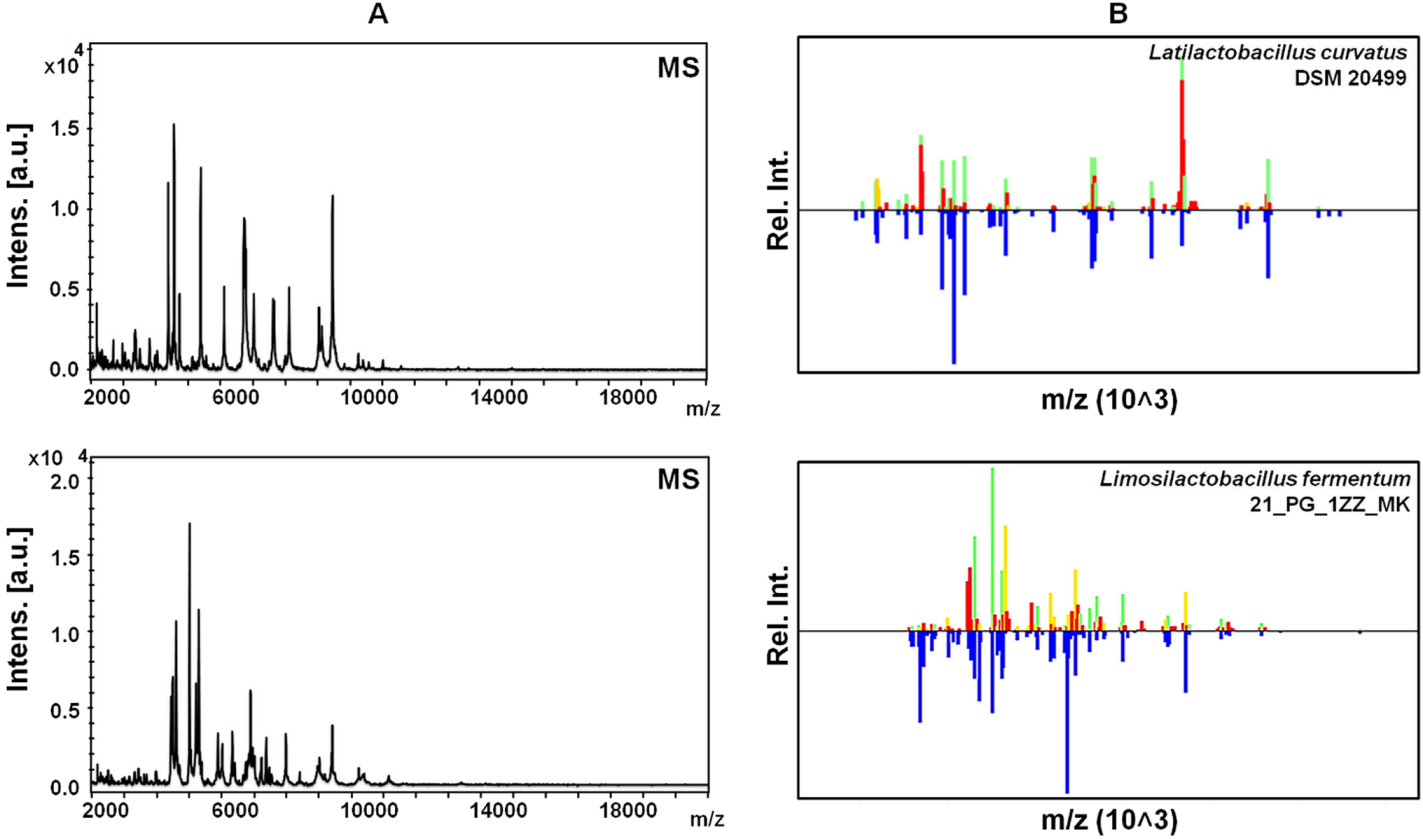
MS (**A**) and MSP (**B**) spectrum showing picklist of the *Latilactobacillus curvatus* DSM 20,499 and *Limosilactobacillus fermentum* 21_PG_1ZZ_MK reference strains identified by MALDI Biotyper Compass platform. Green color – signal of the sample match with reference specter in probability higher than 85%, yellow – signal of sample match with probability lower than 85%, red – no match

Identification of Lactobacilli strains has been achieved using the MALDI Biotyper Compass platform based on the Raw and Main Spectra (MSP) instrument-equipped platform of MALDI-TOF/TOF-MS system. Based on the MSP the sequences of our interest overlap with the *Latilactobacillus curvatus* DSM 20,499 and *Limosilactobacillus fermentum* 21_PG_1ZZ_MK profiles and the similarity of the investigated strains was found to be > 1.7; 2.300 for *Latilactobacillus curvatus* and 2.000 for *Limosilactobacillus fermentum*, classified as a high confidence level (Fig. 2). Then, the both isolates have been deposited, under deposit no. B/00399 (*Latilactobacillus curvatus*) and B/00400 (*Limosilactobacillus fermentum*), in the Polish Collection of Microorganisms (PCM).

### (Bio)silver composites characterization

Nanotechnology requires measuring, monitoring, assembling and mediating at Nano scale by involving interdisciplinary approach for the structure, composition and stability characterization as well as medical area application [1]. The synthesis of silver nanocomposites via biological method ((Bio)NCs) seems to be a simple and viable alternative to chemical and physical ones since it still refers to green chemistry. However, such environmentally friendly methods may also encounter limitations concerning the reproducibility process. For instance, simultaneously production of the silver chloride nanoparticles (AgNCl) and metallic silver nanoparticles (AgNPs—metallic silver) can lead to a confused characterization of the respective nanoparticles which consequently can affect the presented interpretation and conclusion. In view of this statement, the complete and adequate characterization appears to be a crucial step in (Bio)NCs; standing as a precaution when the researchers design the instrumental approach for a complete and correct interpretation. Moreover, biological methods often involve the synthesis of nanocomposites containing, besides silver core, naturally coated organic surface which is relatively poorly taken in consideration during the characterization step. Therefore, in the present research we have been taken in consideration the interdisciplinary approach; once all the three types of nanocomposites have been obtained were subjected to the multi-instrumental analysis - microscopy, spectroscopy, spectrometry and Thermogravimetric methods. The employment of different techniques was a key to gain complimentary and particular description of each of them.

**Fig. 3:**
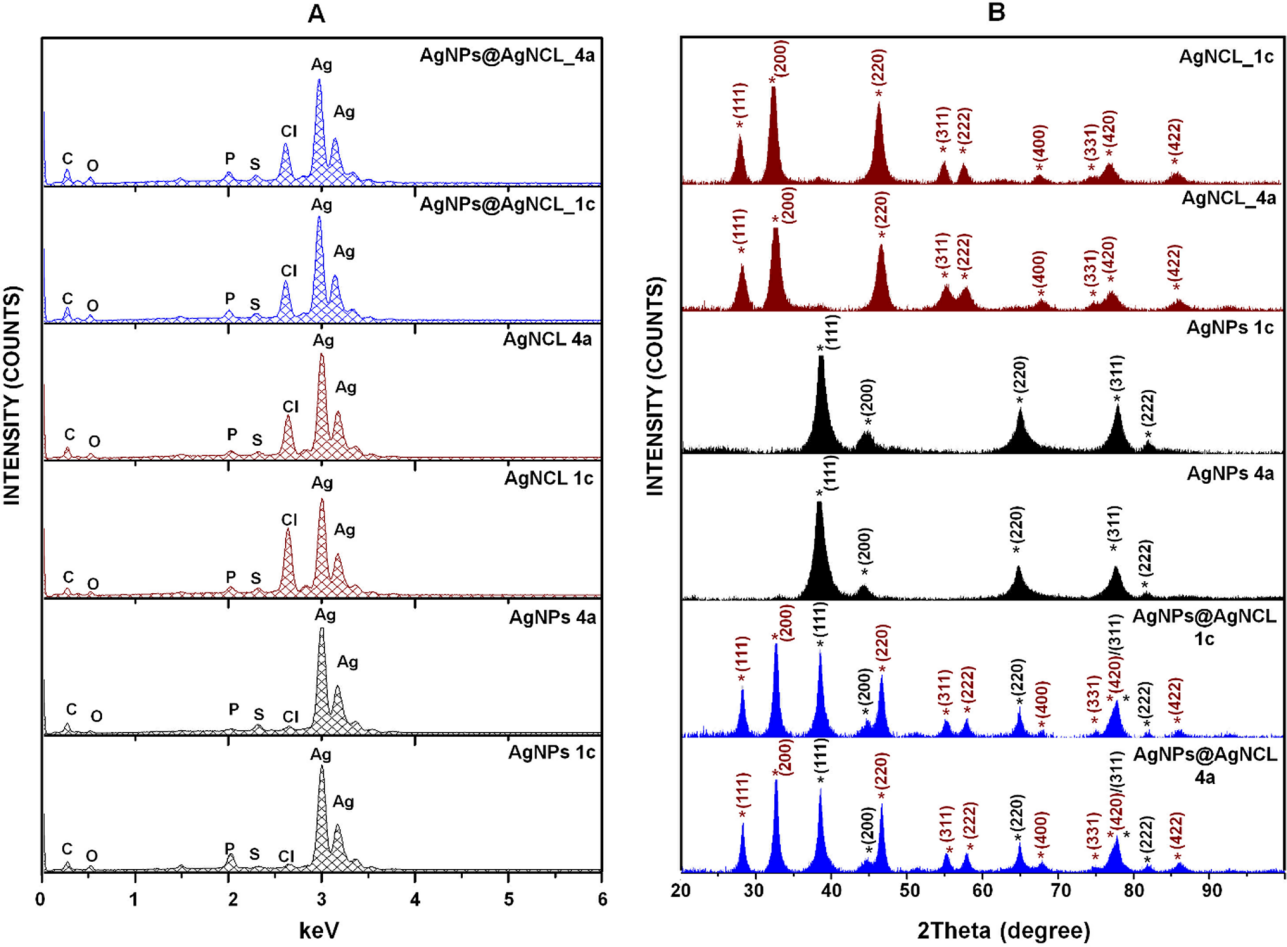
EDX spectrum presenting the elemental composition (**A**) and XRD pattern of all synthesized (Bio)NCs formulations (**B**)

Firstly, all the samples have been investigated for the elemental composition. Figure 3A shows the presence of the silver in the range of 2.7–3.2 keV whereas the binding energy of bio compounds comes from the bio nanocomposites composition. In addition to that, an intense signal characteristic for the chloride has been noticed in the AgNCl and AgNPs H compared to the AgNPs nanoparticles synthesized by both bacterial strains. The additional recorded signals corresponding to the elemental carbon, oxygen, phosphorus and sulfur are the elements that consist of the organic surface coating the silver nanoparticles.

The presence of the chlorides and crystalline nature in the Ag-NCl and Ag-NPs H samples, mediated by the both used strains, was also proven by XRD analysis. Also, the recorded XRD pattern has proven the differences between Ag-NPs and Ag-NCl; recording 5 scattered angles for Ag-NPs, 9 for Ag-NCl and 13 for Ag-NPs H (Fig. 3B). According to the International Crystal structure Database (ICSD) standards Ag/XRD – 64,994 and AgNCl/XRD – 64,734 [9] the exhibited diffraction signals at 2*θ* = 38°, 44°, 64°, 77°, 81° are indexed to the Ag-NPs (111), (200), (220), (311), (222) planes, whereas the signals located at 2*θ* = 27°, 32°, 46°, 54°, 57°, 67°, 74°, 76°, 85° correspond to the (111), (200), (220), (311), (222), (400), (331), (420), (422) planes of Ag-NCl.

For the Ag-NPs H hybrid (AgNPs/AgNCl) samples, the XRD pattern includes both the diffraction signals indexed to the Ag-NPs as well as to the Ag-NCl planes. The noticed signals recorded at 2*θ* = 27°, 32°, 38°, 44°, 46°, 54°, 57°, 64°, 67°, 74°, 76°, 81°, 85° are attributed to the (111) Ag-NCl, (200) Ag-NCl, (111) Ag-NPs, (200) Ag-NPs, (220) Ag-NCl, (311) Ag-NCl, (222) Ag-NCl, (220) Ag-NPs, (400) Ag-NCl, (331) Ag-NCl, (311)@(420) AgNPs@AgNCl, (222) Ag-NPs, (422) Ag-NCl, respectively. Moreover, based on the Bragg's equation, the d-spacing calculated from the first signal, was found to be 0.234 nm for AgNPs and 0.329 nm for Ag-NCl and AgNPs H samples. The obtained results indicate that the biologically synthesized nanoparticles are mediated as an crystalline structure of AgNPs, AgNCl and in case of Ag-NPs H as a hybrid of AgNPs@AgNCl [9]. The XRD technique is a well-known as a powerful analysis for the identification of the material based on the scattered angles of the X-rays that leave the material.

**Fig. 4:**
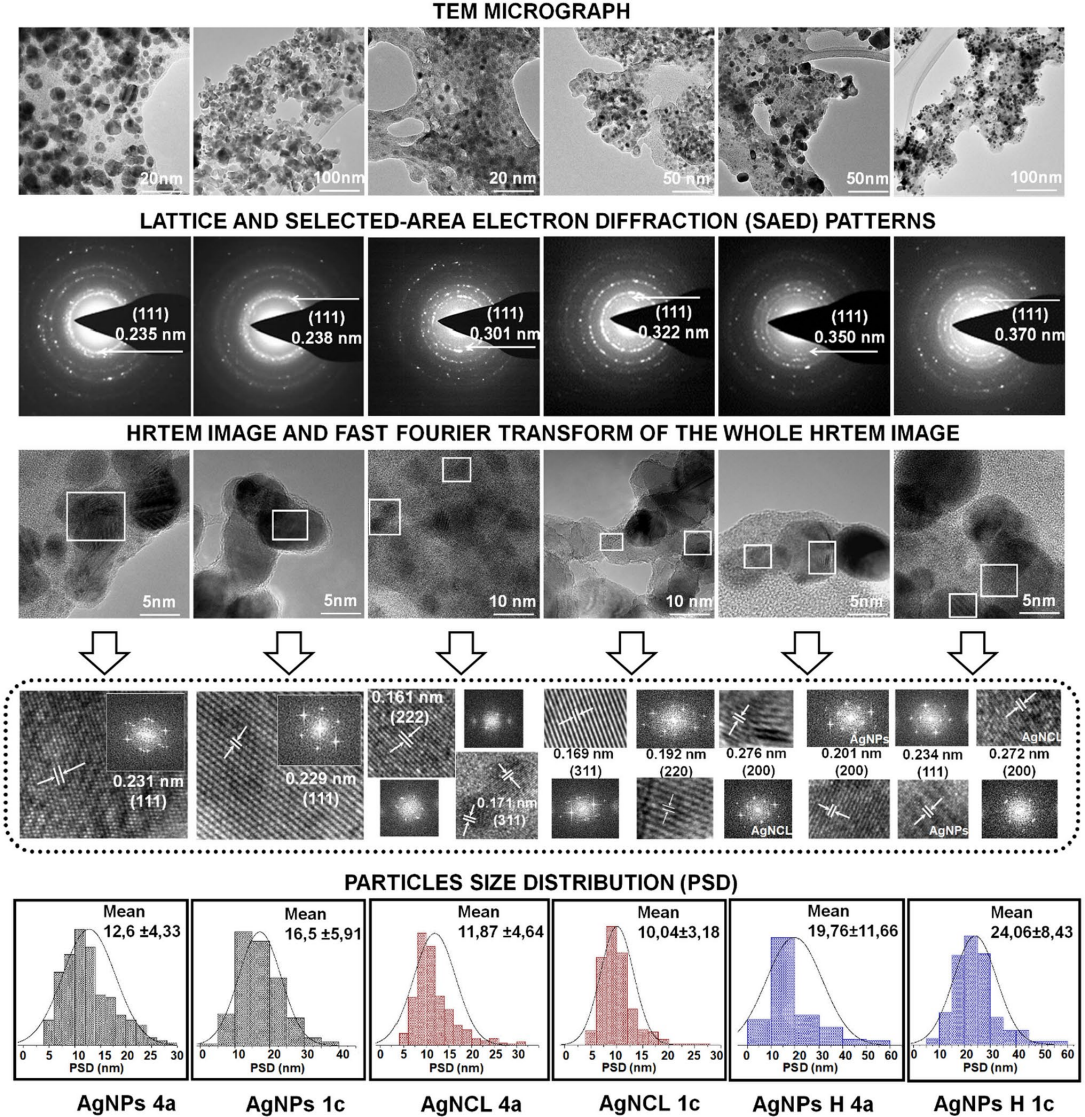
Illustration of TEM micrograph, lattice and selected-area electron diffraction (SAED) patterns, HRTEM image and Fast Fourier transform of the whole HRTEM image showing lattice reflections and particle size distribution of the all synthesized nanoparticles

The diffraction signals of the AgNPs, AgNCl and AgNPs H samples, synthesized by both bacterial strains are similar. Moreover, no additional signals were noticed in the diffraction pattern of each sample, indicating the formation of pure crystalline structure of all obtained nanoparticles, proving also the efficient separation of AgNPs and AgNCl nanoparticles. This phenomenon has also been confirmed by TEM analysis. The Fig. 4 shows the TEM micrograph, lattice and selected-area electron diffraction (SAED) patterns, HRTEM image and Fast Fourier transform of the whole HRTEM image showing lattice reflections and particle size distribution of the all synthesized nanoparticles representing the complex information regarding the differences between pure AgNPs, AgNCl and hybrid of AgNPs@AgNCl (AgNPs H).

The TEM micrograph shows that nanoparticles are homogenously dispersed on the organic matrix. The dispersion was noticed to be comparable for the AgNPs, the AgNCl and AgNPs H synthesized by both bacterial strains. Furthermore, the size distribution of each samples has been measured. The mean of size distribution of each type of synthesized nanoparticles, by measuring the diameter of 230–350 particles, was found to be as follow: 12.6 ± 4.33 (AgNPs 4a), 16.5 ± 5.91 (AgNPs 1c), 11.87 ± 4.64 (AgNCl 4a), 10.04 ± 3.18 (AgNCl 1c), 19.76 ± 11.66 (AgNPs H 4a), 24.06 ± 8.43 (AgNPs H 1c). The presence of nano-agglomerates (clusters) have also been noticed. The size range of nanoparticles synthesized by two different bacterial strains was recorded between 5 and 30 nm for AgNPs, AgNCl and 0–60 nm for AgNPs H (AgNPs@AgNCl) (Fig. 4). In case of the AgNPs H, the predominate population was established to be around 20 nm, showing a higher size in comparison to the AgNPs and AgNCl. However, the selected area electron diffraction (SAED) patterns have proven the crystalline nature of the all synthesized types of nanoparticles. The first concentric diffraction rings correspond to the presence of (111) lattice showing the *d*-spacing value characteristic for the AgNPs (0.235 (4a), 0.238 (1c)), AgNCl (0.301 (4a), (322) (1c)) and AgNPs H (0.350 (4a), 0.370 (1c)) (Fig. 4). Moreover, the corresponding FFT image spot array are indexed for the characteristic lattice plane for each synthetized type of nanoparticles. The recorded Inverse Fast Fourier Transition (IFFT) image show the lattice fringes with the *d-*spacing 0.230 nm for the AgNPs type, which correspond to the (111) plane of silver, whereas the recorded distance between atoms planes for the AgNCl type was found equal to 0.161 nm, 0.171 nm, 0.192 nm which are indexed as a (222), (311), (220) plane of silver chloride, respectively. In turn, for the hybrid synthesized nanoparticles (AgNPs H), the lattice fringes show the *d*-spacing characteristic for both AgNPs as well as AgNCl type. The 0.201 nm, 0.234 nm values of the distance between atoms are assigned to the (200), (111) AgNPs planes while 0.276 nm and 0.272 nm to the (200) AgNCl plane (Fig. 4).

**Fig. 5:**
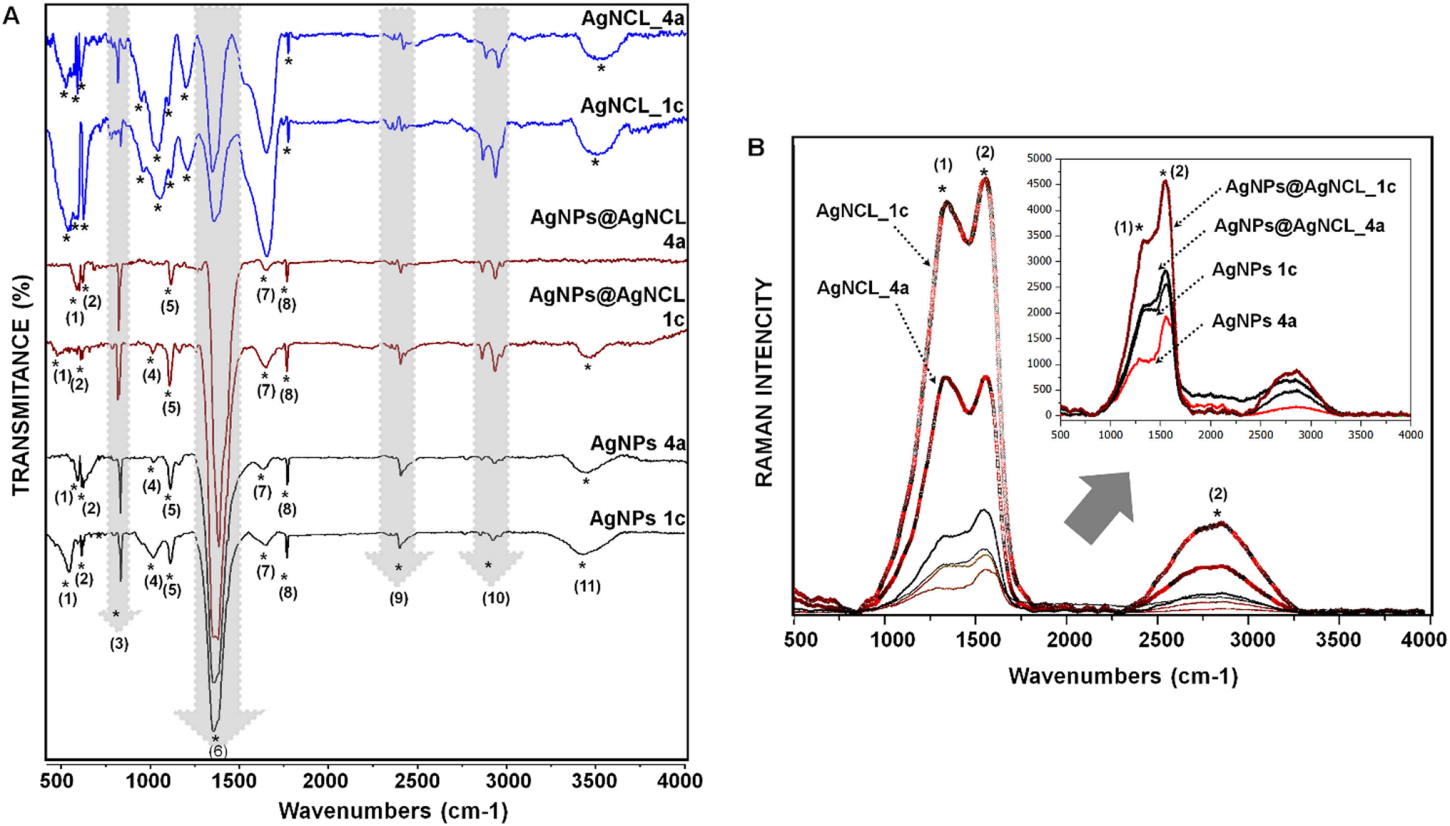
FTIR (**A**) and RAMAN (**B**) spectra presenting discrepancies between AgNCl and AgNPs, AgNPsH types regarding the organic surface

Besides the basic analysis of the structure, size and metallic nanoparticles formation, another important aspect to be taken into consideration is the characterization of the obtained (Bio)nanomaterial is the presence of organics in the (Bio)nanocomposites structure. Moreover, that EDX analysis has initially proven this aspect. Although other techniques including EDX, TEM and XRD are important, the Fourier transform infrared (FTIR) and RAMAN analysis occupies a crucial place as a complementary technique to the adequate characterization of obtained (Bio)NCs. Therefore, the nanoparticles of three types AgNCl, AgNPsH and AgNPs synthesized by two different strains (1c) and (4a) (AgNCl 1c/4a, AgNPsH 1c/4a, AgNPs 1c/4a) were additionally characterized by spectroscopic methods to identify the presence of possible vibrations attributed to the functional groups involved in coating of metallic silver core. The FTIR spectra registered in the range of 500–4000 cm^−1^ are shown in Fig. 5A and suggest the presence of conformation of the registered functional groups mostly in the range characteristic for amide I and amide II vibration (1350–1870 cm^−1^), associated with the binding and stretching vibration directly related to the backbone conformation.

The FTIR spectra illustrate the dissimilarity between AgNCl and AgNPs, AgNPsH types whereas similarity between the nanocomposites (AgNCl 1c/4a, AgNPsH 1c/4a, AgNPs 1c/4a) synthesized by different *Lactobacilli* strain. The intensity of the signals registered in the range 2000–4000 cm^−1^ make the differences between AgNCl and AgNPs, AgNPsH types (Fig. 5A). The broad signals with medium intensity shown in the AgNCl nanocomposites types indicate higher amounts of the respective functional groups in the chloride samples whereas decreasing of them in case of AgNPsH types, followed by those of the AgNPs was noticed. The strong differences have been noticed in the range 500–2000 cm^−1^. The strong and broad bands fall in this range in case of AgNCl nanocomposites types compared to the AgNPs, AgNPsH types. What is more, all registered signals, expected the thin strong signal registered at 1358 cm^−1^, decrease in intensity and being narrow in the spectrum recorded for the AgNPs, AgNPsH types; drastic decreasing of the 1635 cm^−1^ signal and strong increasing of 1358 cm^−1^ was noticed. Moreover, new strong broad bands appeared in FTIR spectra in case of AgNCl type nanocomposite. No differences have been observed for the FTIR spectra generated by the AgNPsH and AgNPs types and slightly dissimilarities between the types synthesized by two different *Lactobacilli* strains. According to the literature data, the respective bands have been identified as a stretching and binding vibration generated by the single, double and triple bond taking place in amino acids structures coming from the organic coats covering the silver core. Accordingly, the signal at 3459 cm^−1^ indicates N–H stretching vibrations that are the source of the amide band, which is part of a Fermi resonance doublet whose frequency depends on the strength of the hydrogen bond. [10]. The signals registered at 2961, 2925 and 2852 cm^−1^ correspond to the stretching bands generated by the C–H aliphatic group [11]. The 1766 cm^−1^ signal [10] indicates the presence of a C=O group of protonated carboxyl groups while the signal found at 1653 cm^−1^ is attributed to the C=O groups of glutamine (Gln) and asparagine (Asn), which overlaps with the absorption of amide I of the polypeptide backbone. The signal 1653 cm^−1^, absorbed in the amide I region is derived from C=O stretching vibrations with a small contribution from C–N out-of-phase stretching vibrations, CCN deformation, and N–H in-plane bending [12]. This signal may also originate from vibrational stretching of CN3H5^+^ arginine (Arg). The absorption signals located at 1358 and 1205 cm^−1^ are associated with tryptophan (Trp) from vibrational stretching of CC [12]. The new signal at 1261 cm^−1^ found only in the AgNCl type nanocomposite originates from C=O and C–C vibrational stretching of tyrosine OH while signals 1169 and 619 cm^−1^ originate from Tyr-OH as resulting from in-plane bending vibration of COH [12]. The bands registered at 1358 and 1261 cm^−1^ also belong to the amide III region (1220–1500 cm^−1^) resulting from N–H bending and C–N stretching [13, 14] and the signal 1169 cm^−1^ is generated due to the presence of glutamic acid (Glu) and aspartic acid (Asp) (stretching vibration C–O). The registered signal at 834 cm^−1^ is caused by the stretching vibration C–C methionine (Met) [12] and for signal 628 cm^−1^ the plane ring deformation modes from phenylalanine (Phe) are observed [15].

The stretching vibrations presence of amino acid residue has also been recorded and proven by RAMAN technique (Fig. 5B). The results show strong differences between AgNCl, and AgNPsH and AgNPs types indicating discrepancies in branch size of surface organic deposit. These summaries were supported by the appearance of the bands showing two prominent signals as a doublets at 1337 and 1559 cm^−1^, respectively, and a broad signal at 2834 cm^−1^ with lower intensity. In fact, the shape of the bands regarding the recorded doublets in the AgNCl types samples shows a strong evolution in case of AgNPsH and AgNPs types; firstly, the decreasing in intensity, secondly, the shift of the 1337 cm^−1^ toward lower intensity from the 1559 cm^−1^, compared to AgNCl types samples, indicating slightly changes in the organic coats composition in the respective samples. Moreover, the obtained Raman spectra indicate repeatedly indicates the similarity between AgNPsH and AgNPs types both in the shape of the obtained signals and their intensities as was confirmed by FTIR analysis. Therefore, the registered signals at 1337 and 1559 cm^−1^ that correspond to symmetric and asymmetric C=O stretching vibrations of the carboxylate group [16] support those affirmations. Additionally, the 1337 cm^−1^ signal also is associated with bending Cα–H vibration in the main-chain of α-helix also entering the region of amide III (1240–1350 cm^−1^). In contrast, a 1552 cm^−1^ signal was also assigned for bands of tryptophan (Trp) residues (indole rings) [17, 18]. The C–H stretching vibrations recorded at 2834 cm^−1^ are attributed to the aliphatic side-chains [18].

In particular, the vibration spectroscopy has as major objective the analysis of the molecular vibration and therefore, the shift of vibration frequencies in case of the biologically synthesised nanocomposites depends on both type of bacterial strains (*Streptacidiphilus* and *Lactoccocus lactic, Lactobacillus paracasei* strains) used for the proper synthesis of the biocolloids [2, 7, 19, 20] as well as of the pH and affinity of the functional groups to the system, e.g. various solvents [2, 20] Many researches, including our studies, have been reported the metallic NPs synthesis as a complexity consisting from the organic matrix represented by the naturally secreted biomolecules outside the cell (e.g. proteolytic enzymes) [21–24].

**Fig. 6:**
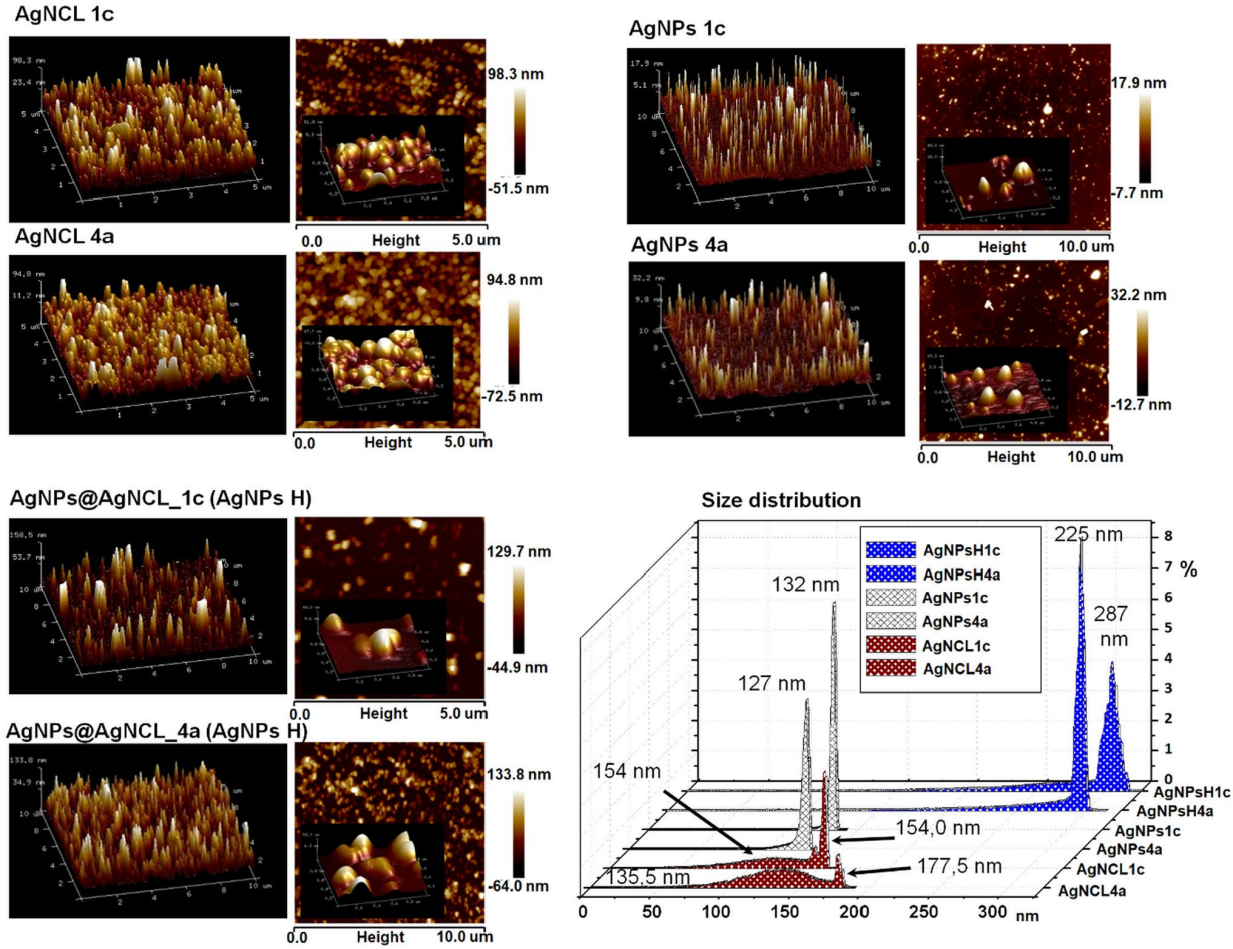
AFM 2D and 3D imaging illustrating topographic information regarding the surface structure and the evaluation of the roughness for all mediated (Bio)NCs types

Among the spectroscopy method, the microscopy approach (AFM microscopy) enables the prediction of topographic information regarding the surface structure and the evaluation of the roughness. Figure 6 presents the size distribution and AFM 2D and 3D imaging showing both the vertical and lateral distribution of height that can be considered in the evaluation of the roughness. Six types of nanocomposites have been imaged and compared by AFM at the same time. All investigated samples visualized by AFM have shown heterogeneity in surface morphology with heterogeneous nanocomposites size. The surface roughness and grain sizes are comparable among all investigated types of nanocomposites while dissimilarity has not been noticed between nanocomposites types (AgNPs, AgNCl and AgNPs H) synthesized by two different bacterial strains.

The AgNCl nanocomposites display round-grain morphology whereas AgNPs types—nanotips-like morphology. The AgNPs H (hybrid) types reveal two topographies: round-grain and nanotips-like area. On the hybrid systems surface, the topographical images visualized the grain layer slightly larger. Moreover, the enlarged grain size shown at higher magnification (1 µm) revealed different cross-section height profiles.

Such quantification and statistical data recorded from the each topographic profile, show the comparison of the equivalent diameters of a grain calculated from the full width at half maximum (FWHM) as follow: 132, 127 nm for AgNPs 1c and AgNPs 4a, 154, 177 nm for AgNCl 1c and AgNCl 4a, 225, 228 nm for AgNPs H 1c and AgNPs H 4a, respectively. The AgNCl type nanoparticles have presented two size populations, the second population recorded the diameter size around 147 nm for the AgNCl 1c and 135 nm for AgNCl 4a, however, not predominant one.

Note that width at half maximum was comparable with the average size of nanoparticles recorded by TEM analysis. The high resolution (HRTEM) micrographs recorded from the dotted area, besides agglomerates show also the presence of twin and multiple twinned particles. The noticed multiple twinned particles suggest the Ostwald ripening phenomenon at initial stage, then, continued to transform and produced silver nanoparticles with larger nanoparticles at next stage [25]. Karkin et al. [26] described the mechanisms of multiply twinned particle formation size during the two-particle agglomeration of the metal clusters, based on the molecular dynamic approach. The authors demonstrate the direct interaction of surface atoms near the point where two particles are touching as a result of the radial distribution function of the central part of one crystal and the amorphous-like region (neck region) allowing the formation of twin-like boundaries with their crystal part. Such reconstruction is promoted by the higher atomic mobility of amorphous-like regions and suitable particle disorientation. On the other hand, it is inferred that the topographical images visualized grain layer consisting of metallic nanoparticles and amorphous matrix, meaning that the organic deposits are naturally coating the silver core. This phenomenon has been proven by DLS measurements where the coated silver biocolloids indicated the average size almost similar to AFM results and higher size value compared to TEM analysis. The DLS analysis measures the distribution of the particles based on the particles width in dynamic fluctuation. The light scattering intensity caused by the Brownian motion of the particles gave the average of the hydrodynamic radius and polydispersity index (PDI) of the particles that generate the degree of uniformity of the samples. According to the International standards organizations (ISOs) regarding the metal silver nanoparticles the PDI value < 0.7 are considered to be a monodispersed system leading to less aggregation while > 0.7 are considered to be polydispersed that can create aggregation. Moreover, it has also been specified that the PDI is influenced by the concentration of the system; higher concentration leads to the formation of the aggregates and as a consequence, to an increase of the PDI value. It has also been noticed that higher concentration provides higher intensity of the light scattered. Once the light scattered is proportional to the diameter to the sixth power, therefore, a population of large particles in the sample can skew the results more than a population of small particles.

**Fig. 7:**
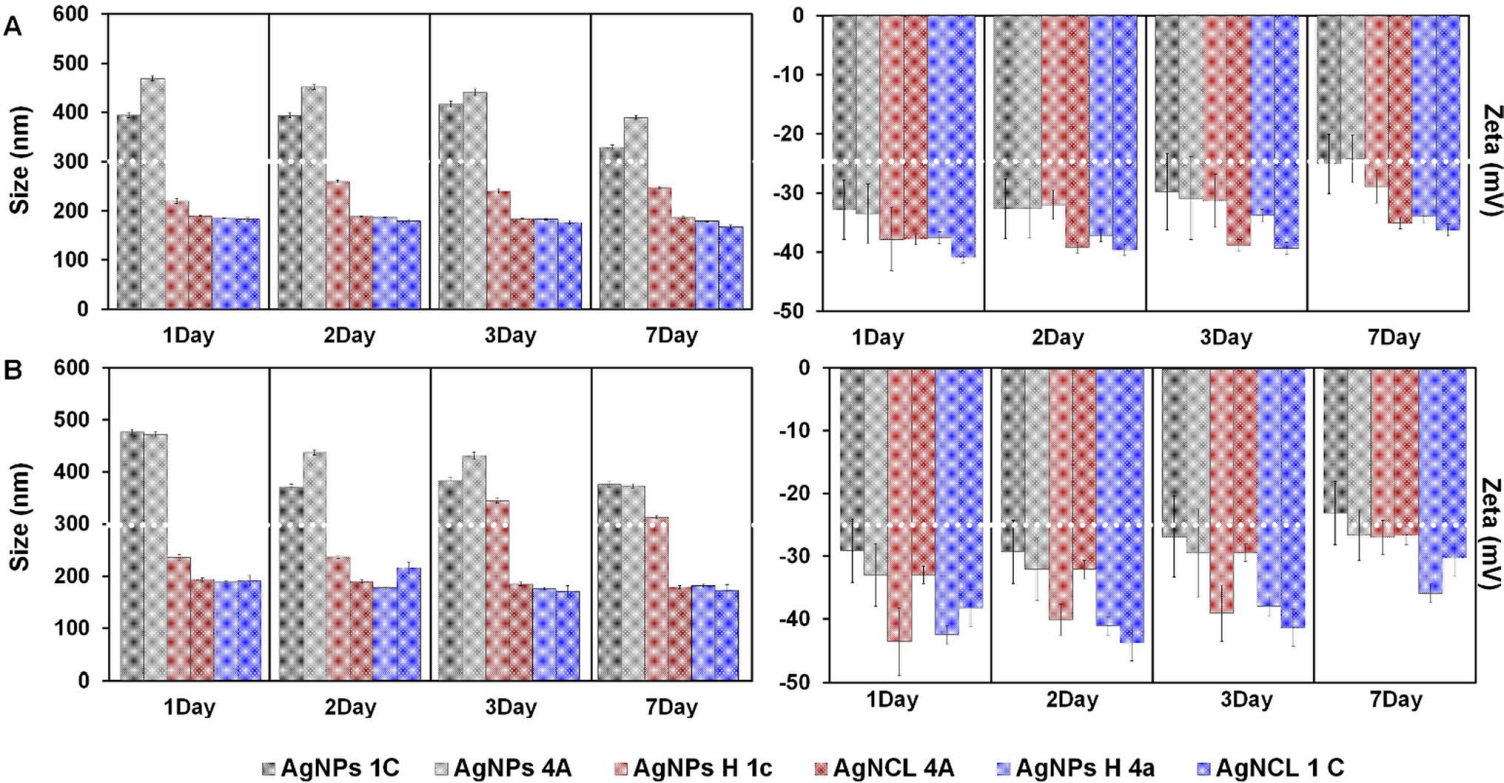
A profile of Size and Zeta potential values presenting the hydrodynamic diameter and the stability of the all synthesized (Bio)NCs types during 7 days at 12.5 µg/mL (**A**) and 25 µg/mL (**B**)

Since the size also influences the stability of the system, and subsequently it is also influenced by concentration, the zeta potential measurement has also been performed. For this reason, for the zeta and size measurement the concentration that was received from the MIC assay (data not shown) has been respected. Considering the fact that MIC value was found to be predominantly in the range of 12.5 (Fig. 7A) and 25 µg/Ml (Fig. 7B), the seta and size measurements have been performed for both concentrations. Moreover, the samples have been monitored measuring after day 1, day 2, day 3, day 7. All tested types of nanocomposites were found to have hydrodynamic diameter ranging from 176 to 480 nm, zeta potential value from − 23 to − 41 mV and PDI from 0.2 to 0.5. As shown in Fig. 7 higher hydrodynamic diameter was recorded for AgNPs types (around 400 nm) whereas for the AgNCl and AgNPs H types, the hydrodynamic diameter was found < 200 nm. A slightly increased diameter was noticed for AgNPs H 1c (260 nm).

No drastic differences were observed between the all investigated nanocomposites regarding the used source for the synthesis (means bacterial strains). Moreover, no drastic changes in hydrodynamic radius was recorded during 7 days of monitoring while in the case of zeta potential a slightly decreasing zeta value was observed. The same trend of decreasing was noticed in case of all investigated samples. However, the zeta potential value was included in the range considered to be stable and even after the 7 days the system was still stable indicating strong repulsion forces between the NPs; suggesting prevention of the agglomerates formation. Similar activity was noticed in case of higher concentration (25 µg/mL) regarding the hydrodynamic radius and zeta potential value. Exception being the AgNPs H 1c which present a slight increase in diameter (from ~ 200 to ~ 300 nm) and a slight decrease in zeta potential (from − 40 to − 30 mV) value after day 3 and day 7.

Insignificant decrease in the stability of the system after day 7 has also been noticed for AgNPs 1c (25 µg/mL) compared to AgNPs 1c (12.5 µg/mL). However, in both cases, all tested samples remained stable after day 7. Moreover, DPI value has been found to be almost the same in both cases (0.2 or 0.3) even after day 7, suggesting that these samples have an acceptable level of uniformity. The intensity distribution of three repetitions in case of all analyzed samples was found > 95%, therefore, once the distribution obtained from a measurement is based on intensity and PDI value < 0.5, the all investigates types of NPs are considered monodispersed systems [27].

**Fig. 8:**
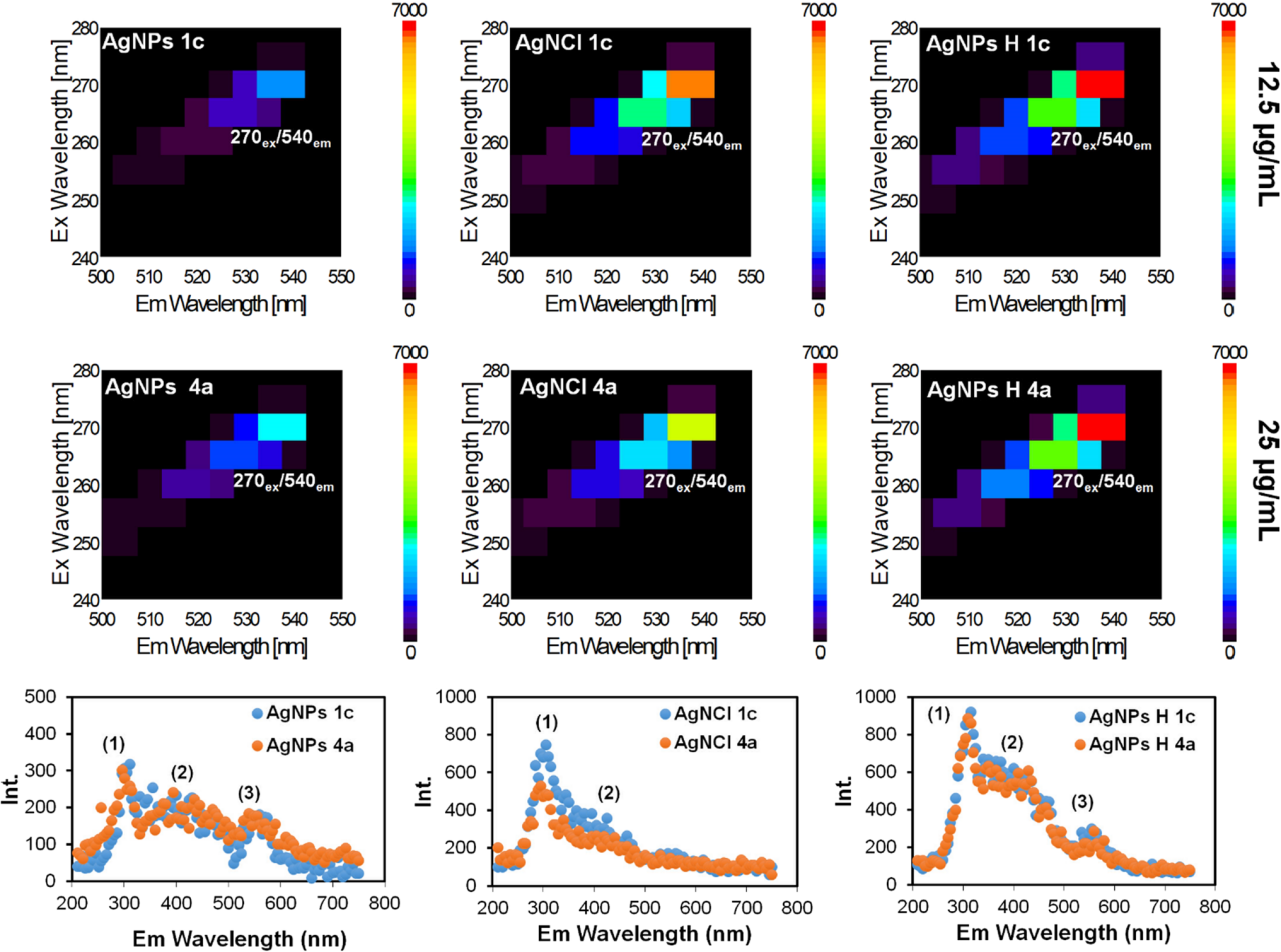
Fluorescence analysis illustrating the fluorescence generated by both silver core and organic deposits recorded in all mediated (Bio)NCs types

The results performed by TEM, DLS and AFM techniques are complementary, generating the detailed characterization regarding the size particle distribution. Therefore, the differences between sizes is related to the used technique: TEM is referred to the silver core size (metallic silver), DLS – to the hydrodynamic radius of nanocomposites, defined as biocolloids whereas AFM – to the nanoscale grain analysis in 3-dimensional image. According to this, the synthesized nanocomposites consist of a silver core that naturally is coated with biomolecules (e.g. amino acids, peptides etc.). This phenomenon has been also confirmed by Fluorescence spectroscopy analysis. Figure 8 illustrates the fluorescence spectra recorded for three types of AgNPs, AgNCl and AgNPs H representing the fluorescence generated by the silver core (Fig. 8A) and fluorescence emission reflected by the organic core (Fig. 8B).

The results depict the *λ*_ex_/*λ*_em_ wavelength for silver core at 270 nm/540 nm and for organic compounds with the maximum emission wavelength registered at 310 nm, 410 nm and 550 nm for the AgNPs types, 310 nm and 410 nm for AgNCl types while 310 nm, 410 nm and 550 nm for AgNPs H (hybrids).

As shown in Fig. 8B, the emission spectra illustrate the similarity between the nanocomposites mediated by different strains (*1c–L. curvatus; 4a–L. fermentum*) and dissimilarity between each type of nanocomposites (AgNPs, AgNCl and AgNPs@AgNCl) concerning both the fluorescence signals as well as fluorescence intensity.

The intensity of the fluorescence regarding the biomolecules (organic core) of AgNCl and AgNPs@AgNCl was stronger in comparison to the AgNPs types, indicating a large amount of organics in the complex matrix. Additionally, it is important to point out that such biomolecules as amino acids are inherently responsible for fluorescence. The emission spectrum recorded in a range between 200 and 800 nm showed three signal with the maximum intensity for AgNPs, and AgNPs@AgNCl and only two signals for AgNCl types suggesting the fluorescence that corresponds to emission by the aromatic side-chains (Tryptophan, Tyrosine, Phenylalanine) [11] residues in proteins at ~ 300 nm, non-aromatic amino acids (Glutamine, Arginine, Alanine, Cysteine etc.) [28] at ~ 400 nm, whereas peptides residue attached via C-terminus to a central lysine spacer (e.g. Gly–Ser–Lys) at ~ 530 nm [29]. Therefore, the recorded fluorescence originated from both organic interface and metal core. Fluorescence and emission spectra for all three types of synthesized nanocomposites were similar, indicating that the organic shell played a key role in the fluorescence mechanisms and therefore, the fluorescence is attributed to states related to the organic coats - metal interface.

The fluorescence response follows the following order AgNPs, AgNCl and AgNPs H. However, although the recorded spectra for nanocomposites types are quite distinct and easily differentiable, it is essential to specify that the absorptivity of AgNPs and AgNPs H (hybrids) types is an order of magnitude larger than AgNCl types; span the UV and Visible range.

Among the described particularities, which already have shown that the mediated (Bio)AgNCs nanocomposites types consist of a complex structure, containing silver core coated by the organic surface, and taking in consideration the limitation of each technique, a conjunction approach was needed to provide a more complete background. One of the assumptions that was immediately investigated it was the existence of the biomolecules in composition of the mediated nanocomposites types by spectrometric laser desorption ionization (LDI) approach in tandem with time-of-flight analyzer (LDI TOF-TOF MS). Moreover, as a complementary information has also been identified the amino acids and peptides sequences in the organic layer, covering the surface of silver core.

Based on our previous results, [7, 20] the organic layer consists of the metabolites (amino acids and peptides sequences) secreted by the bacterial strains involved in the mediation of the silver nanocomposites. These strategy give a justification that secreted biomolecules bind naturally in/into silver core in different modes and notable to emphasize that different peptides sequences have been recorded for silver nanocomposites synthesized by different LAB strains. Moreover, not only the organic layer but also silver-organic connection and the simple silver isotopes and silver clusters presence have been noticed. Therefore, the results in present study become much more significant when the one- and two dimensional LDI analysis have been performed for all mediated nanocomposites types.

Notable to underline that in current research, concerning the surface plasmon resonance (SPR) properties of metallic nanostructured nanoparticles and their potential to chare donor for themselves and molecules binding on their surface, the LDI analysis were carried out without matrix. Thus, the obtained nanocomposites have also been investigated for their application potential as a matrix such us in nanostructure-assisted laser desorption/ionization (NALDI) [7]. Additionally, the application of FlexControl (referred to the building blocks) and isotopic pattern software based on the LIFT fragmentation results, have identified the amino acids and peptides sequence presence as well as the hill sorted sum formula.

**Fig. 9:**
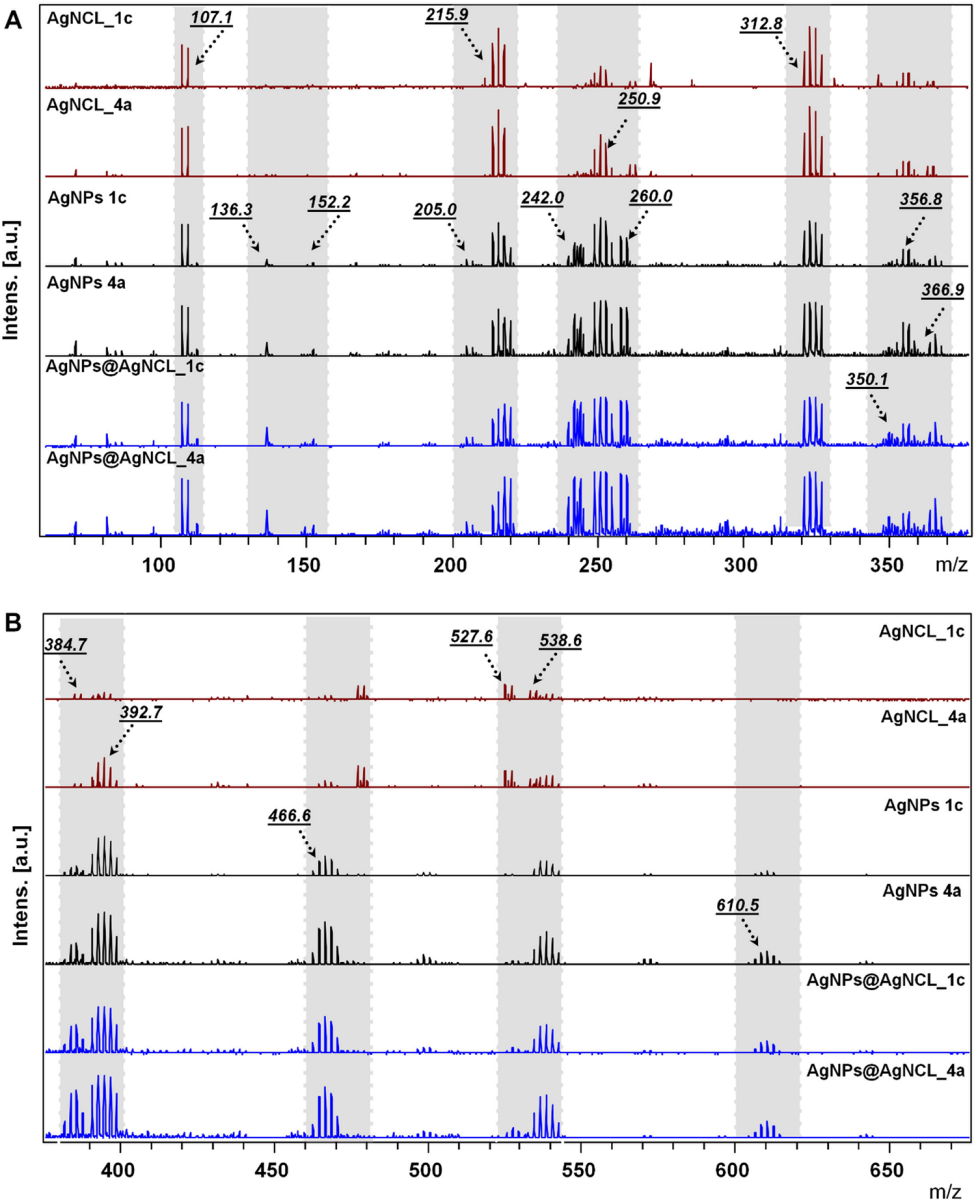
MALDI mass spectra presenting MS view of discrepancies of molecular fingerprint and isotopic distribution for all synthesized (Bio)NCs types. Illustration of one- (1-) dimensional spectra from m/z = 100 to m/z = 350 (**A**) and from m/z = 350 to m/z = 700 (**B**)

Therefore, the Fig. 9 displays one- (1-D) LDI MS spectra of molecular fingerprint of the all synthetized types of nanocomposites (AgNPs, AgNCl and AgNPs@AgNCl) showing the silver isotopes, silver-cluster and silver-organic connection presence whereas Fig. 9A illustrates the two- (2-D) LDI LIFT MS of organic coats and isotopic distribution of selected signals. The signals have been chosen based on the appearance strategy of the recorded signals that shows the discrepancies between AgNCl and AgNPs and AgNPs @ AgNCl types.

**Fig. 10:**
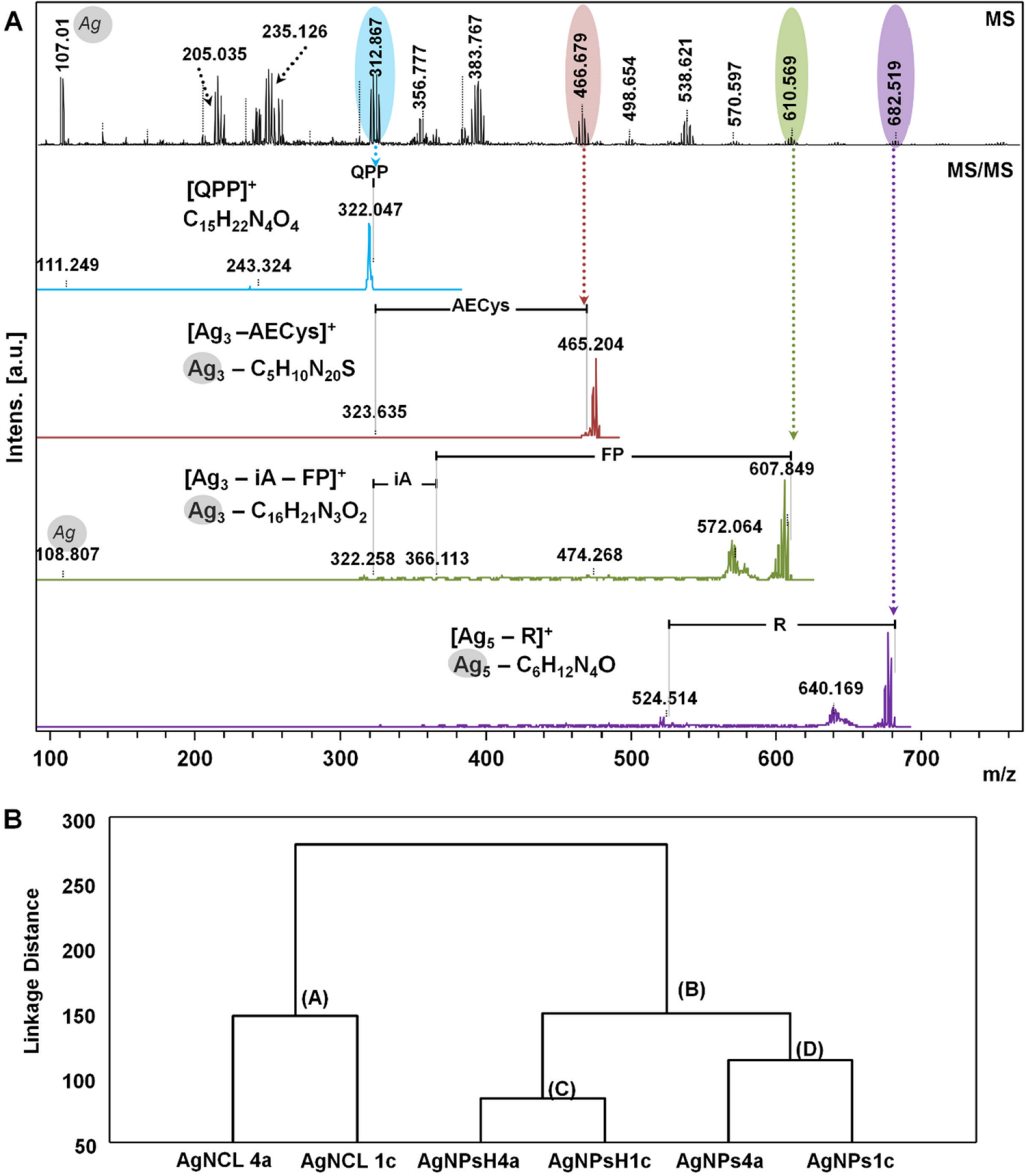
An example of MS/MS spectra **A** of the metal–organic signals and hierarchical cluster analysis **B** presenting dissimilarity based on the (Bio)NCs profiles

The LDI analysis has been performed in a range between 100 and 2000 m/z. To follow the evident differences between the molecular fingerprint of the synthesized silver types, regarding the organic deposits and silver isotopic distributions, the Fig. 9 illustrates the one- (1-) dimensional spectra of AgNPs, AgNCl and AgNPs@AgNCl types from m/z = 100 to m/z = 350 and from m/z = 350 to m/z = 700, whereas > 700 referred to the silver isotopic distributions, are presented in Fig. S1. Evident similarity has been observed between the molecular fingerprint of AgNCl and AgNPs, AgNPs@AgNCl types, especially in the range < 500 m/z. Starting with the m/z > 500, significant molecular fingerprint differences were noticed, indicating the higher SPR potential of the AgNPs and AgNPs@AgNCl types compared to AgNCl types. In the respective region strong silver isotopic distributions was recorded leading to the formation of multi-([Ag]^+^) clusters generated by the different number of atoms (see Fig. S1, m/z = 682.5 [Ag6]^+^, m/z = 754.1 [Ag7]^+^, m/z = 859.0 [Ag8]^+^). In turn, the common signals recorded in the range < 500 m/z, in case of all investigated samples, indicate the evident similarity of the sample not only concerning the silver isotopes (m/z = 107.149 [Ag]^+^, m/z = 215.773 [Ag2]^+^) but also the organic coats (m/z = 322.8 [QPP]) and silver-organic connection (m/z = 465.2 [Ag_3_ – iA – FP]^+^) (Fig. 10A). On the other hand, intensity decreasing (m/z = 250.9; m/z = 366.9; m/z = 384.7; m/z = 392.7; m/z = 465.2; m/z = 538.6 (Fig. 10B); m/z = 682.5 and m/z = 859.0, see Fig. S1), disappearance (m/z = 135.3, m/z = 152.2, m/z = 242.0, m/z = 607.8) or the presence of the characteristic (m/z = 527.6; m/z = 695.1, see Fig. S1) signals in the AgNCl types, prove repeatedly the discrepancies between AgNCl and AgNPs and AgNPs@AgNCl types.

Additionally, it is notable to emphasize that using the model isotopic pattern software, the amount of the Ag in the metal–organic connections (C_5_H_11_Ag_3_N_20_S_1,_ m/z = 465.2; C_16_H_22_Ag_3_N_3_O_2_, m/z = 607.8 and C_6_H_13_Ag_5_N_4_O_1_, m/z = 640.1) was found to be in the range of 45.78–77.4% whereas amount of the N was noticed from 6.87 to 39.63% followed by the O (5.23%) and S (4.54%). Therefore, it is conceivable that the respective observations demonstrate the fact that isotopic pattern of silver (d-electrons metal) involves the binding of the organics coats to ions and clusters by the N, O, S elements coming from the structure of the identified amino acids structure naturally located in the surface coats in/onto silver core.

Furthermore, the Fig. 10B illustrates dendrogram representing the statistical differentiates of the investigated silver nanocomposites types based on the obtained LDI MS profiles. A dendrogram presents the formation of two separated categories of clusters: giving the distinguishing firstly between (A) and (B) and then, the (B) cluster present again different cluster modeling (C and D), justifying the strong differences between AgNCl types and AgNPs, AgNPs@AgNCl types whereas similarity between AgNPs@AgNCl and AgNPs. However, no differences of molecular fingerprint and silver isotopic distributions between AgNPs and AgNPs@AgNCl types was noticed not discrepancies between the nanocomposites types synthesized by different LAB strains, justifying that those types of nanocomposites synthesized by 2 different *Lactobacillus* strains are very similar with respect to organic coats, silver-organic combinations and silver clusters distribution.

**Fig. 11:**
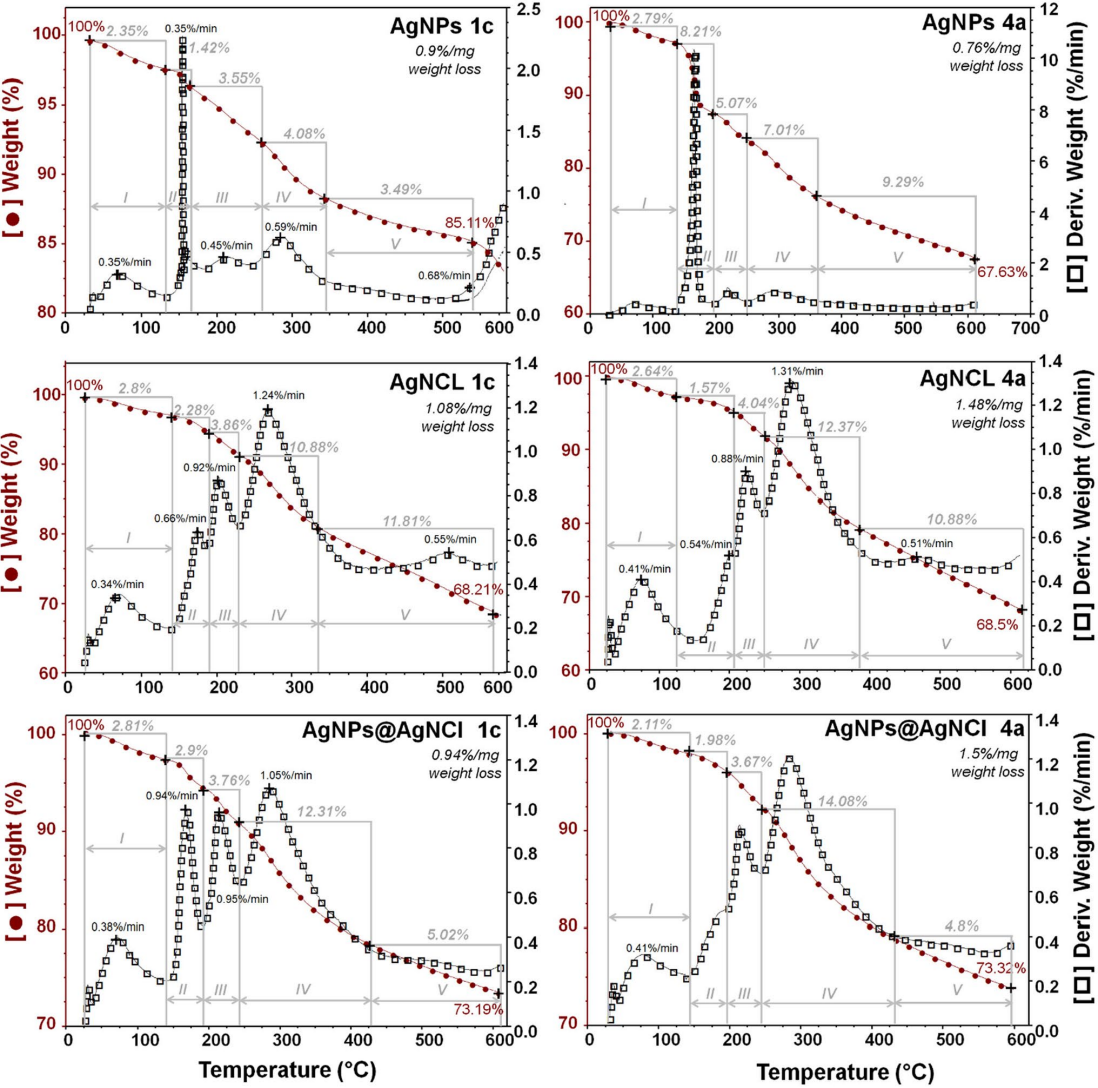
TG and DTG curves recorded in the range of 0–600 °C presenting dissimilarity between (Bio)NCs types

Once the all obtained (Bio) silver formulations incorporate both metallic silver core as well as capped organic surface covering the silver core, thermal stability of the (Bio)silver nanocomposites is required to compliment the already gained characteristics and properties of each type of (Bio)silver nanocomposites. To determine the stability and indicate the nature of the obtained nanocomposites under temperature dependence, thermogravimetric (TG) analysis was carried out and represented by the TG and derivative TG (DTG) curves recorded in the range of 0–600 °C (Fig. 11). The DTG curve shows clear similarity for AgNCl and AgNPsH nanocomposites types compared to AgNPs types, in contrast to FTIR and Raman spectroscopic analyses.

The decompositions of the biomasses employed V stages and the mass loss processes occurred slowly. For all investigated samples the I, II, III and V stages take place in a similar way whereas IV stage in slightly different manner: I stage is characterized by weight loss from 2 to 150 °C (< 3%), II stage to 200 °C, III stage to 260 °C, IV stage to 350 °C for AgNPs types, to 380 °C for AgNCl types and to 450 for AgNPs @ AgNCl types, V stage to 600 °C. Moreover, a dissimilarity of loss mass has been noticed for AgNCl and AgNPs @ AgNCl types compared to AgNPs types at IV stage whereas dissimilarity between AgNCl and AgNPs @ AgNCl at V stage regarding the mass loss rate. For instance, at IV stage, the AgNCl and AgNPs@AgNCl types nanocomposites recorded the higher weight loss around 10–14% with the mass loss rate c.a 1%/min while at V stage AgNPs @ AgNCl – c.a 5% (weight loss), 0.3%/min (mass loss rate), much less than AgNCl types, which have shown higher weight loss (11%) with a mass loss rate 0.5%/min. However, the total weight loss in case of AgNPs @ AgNCl was noticed to be around 27%, not much less than AgNCl (31%). No differences have been observed between the AgNCl and AgNPs@AgNCl types synthesized by different strains.

In turn to AgNCl, AgNPs @ AgNCl nanocomposites types, strong discrepancy has been observed in case of degradation behavior for AgNPs; firstly the differences between the types of (Bio)silver composites and secondly between the AgNPs mediated by different strains, indicating analogy in weight loss process during the degradation process and evident comparison in mass loss rate at II stage; 0.3%/min for AgNPs 1c and 9.5%/min for AgNPs 4a. What is more, the total weight loss in case of AgNPs 1c was found to be around 15% while in case of AgNPs 4a – 25%.

Therefore, the biomass degradation process has proven one more time the presence of organic coats; what is more, the TG results are in strong concordance with the fluorescence results regarding the fluorescence generated by the organics. The initial weight loss at I stage is generated by the degradation of water molecules attached to silver nanocomposites structure followed by the elimination of coordinated water molecules, but also from the degradation of organic molecules covered the silver nanoparticles core at II, III, IV and V [30]. On the other hand, it is supposed that the observations recorded at IV stage can be also a consequence of the degradation of aromatic amino acids such as tyrosine (Tyr), phenylalanine (Phe) and tryptophan (Trp). [31]

Upon these explanations regarding the discrepancies characterization and particularities of AgNPs, AgNCl and AgNPs@AgNCl formulations, the present study is also coming to elucidate the differences of mechanism formations among them.

**Fig. 12:**
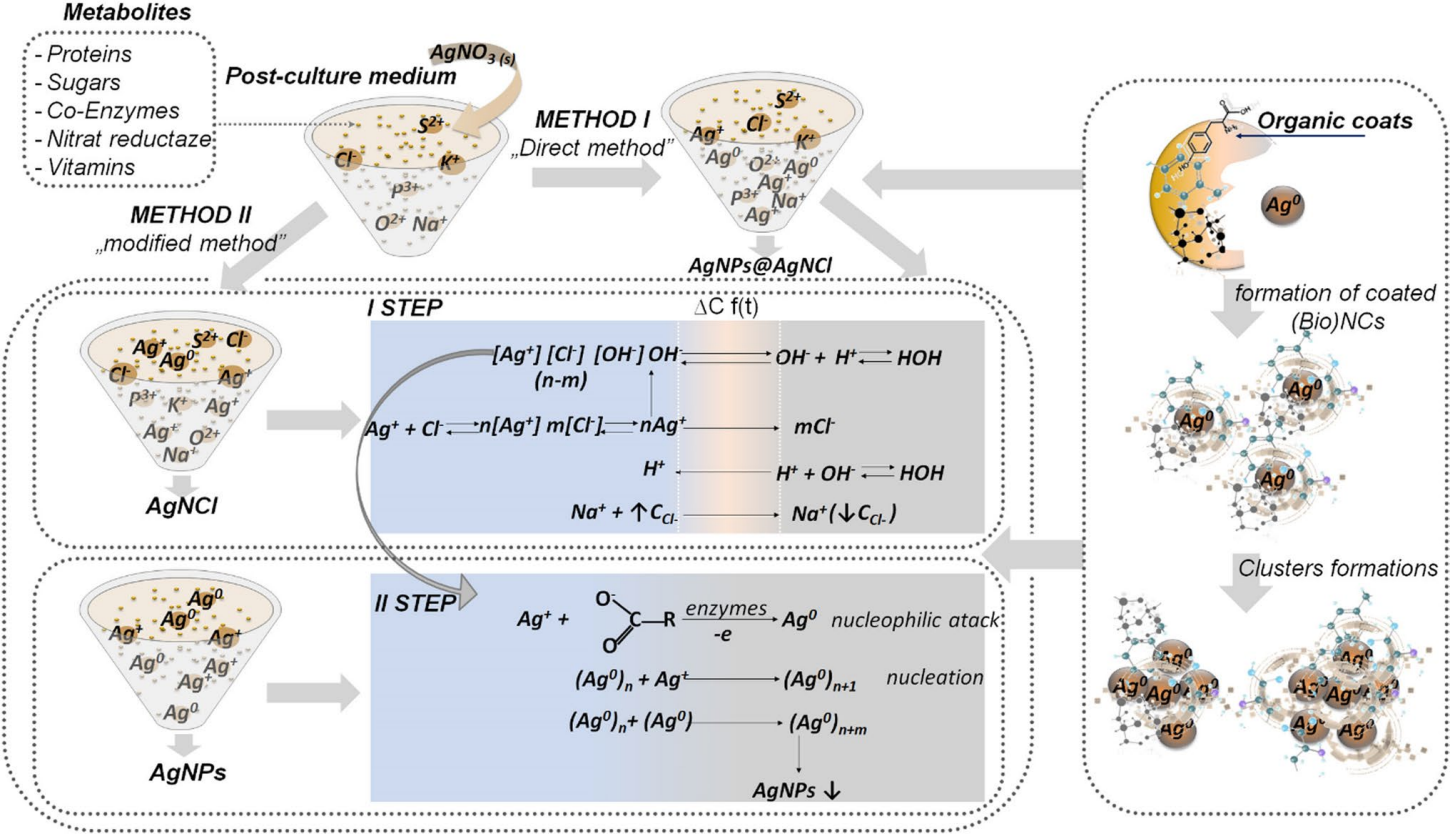
Scheme illustration presenting the mechanism formations of all mediated (Bio)NCs types by “direct method” and “modified method”

Although many studies [32–34] have described the mechanism formation of silver nanocomposites (AgNPs, AgNCl – hybrid form) the current research proposed, besides the deep characterization, the mediation mechanism of all silver types formulations. Therefore, based on our experimental findings, the mechanism formations of all mediated types takes place in different manners (Fig. 12). In case of “modified method” the formation of the (Bio)NCs types occurs in three steps: firstly the formation of AgNCl type takes place (I step) by the salt metathesis reaction [35] which consequently precipitate immediately involving the combining an aqueous solution of silver nitrate (which is soluble) with a soluble chloride salt contained in MRSB medium used for the synthesis – the reaction occurred till the all Cl^−^ ions were completely involved; secondly, Ag^+^ is converted to Ag^0^ via proton transfer reaction with side chain of the amino acid residue, secreted by the used LAB strains – the nucleophilic attack reaction take place continuously and simultaneously with the nucleation stage until the exchange reactions occurred completely. What is necessary to underline it is the fact that the silver chlorides, metallic silver formation and nucleation stages are accompanied by an organic branch covering process. According to the “modified method” the formation of AgNCl and AgNPs takes place separately. In turn, in the case of “direct method” – all the enumerated reactions take place successively in hybrid manner formation.

Moreover, the MALDI results have demonstrated strong silver isotopic distributions leading to the formation of multi-([Ag]^+^) clusters generated by the different number of atoms, indicated both the reduction of silver ions to metallic silver as well as nucleation stage; the presents and identification of the organic functional groups that are involved in mediations mechanism have also been noticed. What is more, the respective affirmations are also supported by all spectroscopy, microscopy methods employed in present study.

Many reports have demonstrated the ability of LAB strains to secret various metabolites [24] and hypothesized the role of them in the reduction of silver ion to metallic silver [33] and consequently formation of multi silver clusters by nucleation process [33, 36]. For instance, hydroxyl groups of tyrosine residues, carboxylic acid-containing peptides, and carboxyl groups of aspartic acid and glutamic acid residues were found to be involved in (Bio)NCs mediation [37].

Moreover, many researches show the possible bacterial strains to produce AgNCl, in the absence of AgNPs; the hybrid form (AgNCl@ AgNPs) have as wee been reported [9]. However, the exact mechanism has not been elucidated. As it was mentioned above the biological method may also encounter a certain limitation concerning the reproducibility process; occurrence of AgNCl is taking place and this aspect may lead to a confused characterization and interpretation of these two (Bio)NCs types (AgNPs and AgNCl), even more, as a consequence, can interfere and can affect the stability or/and antimicrobial activity of obtained formulations. On the other hand, it is noteworthy the fact that the used medium for the growing step of the bacterial strains also play a crucial role. As it is reported by the manufacturer, the composition of the growth medium vary from one species to another and contain various nutrients that adjust and/or supplement as a requested step to meet performance criteria: peptones, extracts that serve as a source of carbon, nitrogen, amino acids, sulphur, vitamins; starch, carbohydrates – serve as a source of energy and salts that maintain the osmotic equilibrium of the medium. Moreover, the Durán et al. [9] summarized many research studies that report the synthesis of both AgNPs and AgNCl types using different growth medium to obtain post-culture medium, as a rich metabolite medium that will be involved in both types of mediation. For instance, the Lysogeny broth medium, T3 medium and Potato dextrose medium lead to the AgNPs formation while the Salmonella Shigella to the AgNCl type; it is notable to specify that Potato dextrose medium has also been found to produce mixed types, means hybrids (AgNPs@AgNCl). It is not ruled out that concentration of used AgNO_3_ plays also a crucial role once in one case the author used 1 mM and in another case 10 mM. The higher concentration of AgNO_3_ led to the formation not only of the AgNPs but also of the AgNCl type. This phenomenon is proved by the well-known inorganic chemistry of silver properties to induce immediately AgNCl in ratio 1:1 and then, reduction of remaining Ag^+^ to Ag^0^ [35]. For what is more, the composition of chloride salts, according to the manufacture, varies from one medium to another, therefore the amount of Cl^−^ will influence the mediated amount of AgNCl and consequently negligible, slight or high amount of chloride type NPs will classify their predominance/subservience in hybrid system. Therefore, in this order of ideas, it is required to underline that not only the used safe source (bacteria or fungi species) will influence the mediation of the hybrid system but also the used medium and silver nitrate concentration. Additionally, the chosen medium for the synthesis is directly correlated to the ability growth of the respective species to achieve the higher amount of the secreted metabolites and consequently, the reproducibility of the synthesis. The chosen medium (MRSB medium) for the present research was related to this aspect once the MRSB medium is suggested by the manufacturer as a dedicated culture medium for the *Lactobacillus strains*. Therefore, the appearance of huge amount of AgNCl during the synthesis using “directed method” and taking into consideration the fact that stability and antimicrobial activity of such (Bio)NCs may be influenced by their presence in the hybrid system and as a consequence the diverse application area, it was attempted to modified the method by separation of AgNCl and AgNPs types. Moreover, to compare how the physico-chemical properties are influenced including also the impact of organic coats, as o comparison, the present research describes the outcomes and related discussion concerning the use of two different *Lactobacillus strains* (1c and 4a) to synthesized three types of (Bio)NCs, means six specimens. In view of these statements, the present study, aimed to indeed shed further light firstly, on promotion of sustainable development and secondly, summarize the discrepancy of the AgNPs, AgNCl and their hybrid (AgNPs, AgNCl) concerning complete characterization including even mediation mechanisms. Besides the detailed and relevant characteristics (properties) of each specimen the mechanism formation also differs, suggesting strong discrepancy between the AgNCl, AgNPs and AgNCl@AgNPs types. Furthermore, in light of characteristics discrepancy, it is supposed that the antimicrobial properties impact should be highly considered and an adequate approach is also needed to be designed.

## Patent

The details of both isolation method as well as silver nanoparticles, silver chloride nanoparticles and hybrid silver synthesis method using *Latilactobacillus curvatus*, and *Limosilactobacillus fermentum* mentioned in this study is covered by a patent application in Poland – no. P.441585.

## Conclusions

Since silver nanoparticles have become an attractive field due to the environmentally-friendly approach, adding that this aspect has fast achieved great progress, the designing of an exhaustive and adequate characterization and interpretation of this field is urgently needed; especially when the sustainable development and biological method are involved. Therefore, the present study, for the first time, emphasizes two different methods for the biological mediation (“direct method” and “modified method”) of available and inexpensive novel LAB sources (*Lactobacilli*) using bioactive source (e.g. milk); simultaneously promoting the recycling path (sustainable development) and synthesis and discrepancy of AgNPs, AgNCl and AgNPs@AgNCl nanoparticles types properties via interdisciplinary approach. The present work also seeks to design a new approach onto a general frame on the novel knowledge regarding the biogenic silver nanocomposites as a complimentary interpretation. The use of spectroscopy, microscopy, spectrometry and thermogravimetric analysis allows us to distinguish and present the differences on the characteristics of tree types of (Bio)NCs and also demonstrated one more time that the silver nanocomposites synthesized by biological method represent a complex structure directly linked to the metallic silver core and organic branching coats. Moreover, the spectrometric and spectroscopic studies have proven the presence of the metabolites (e.g. amino acids and peptides sequences) secreted by the used *Lactobacillus strains*. According to these statements and based on the obtained results and interpretation regarding the differences among them, the mediation mechanism of each (Bio)NCs type has been designed suggesting different mediation mechanism for each type (AgNPs, AgNCl and AgNPs@AgNCl) of obtained nanocomposites. In this regard, in order to avoid a misunderstanding of physico-chemical characteristics of AgNPs, AgNCl and AgNPs@AgNCl nanocomposites, it is required to employ a precaution on the characterization step since the AgNCl might be simultaneously mediated.

## Supplementary Information

**Additional file 1**: **Fig. S1**. MS spectrum showing m/z ranged between 600 and 1100

## Data Availability

The datasets generated and analyzed during the current study are available from the corresponding authors on reasonable request.
