# Supplementary figures and images for: Consideration of a new approach to clarify the mechanism formation of AgNPs, AgNCl and AgNPs@AgNCl synthesized by biological method
Source: Discov Nano. 2023 Feb 1;18(1):2. doi: 10.1186/s11671-023-03777-w (PMC9892381; doi:10.1186/s11671-023-03777-w)

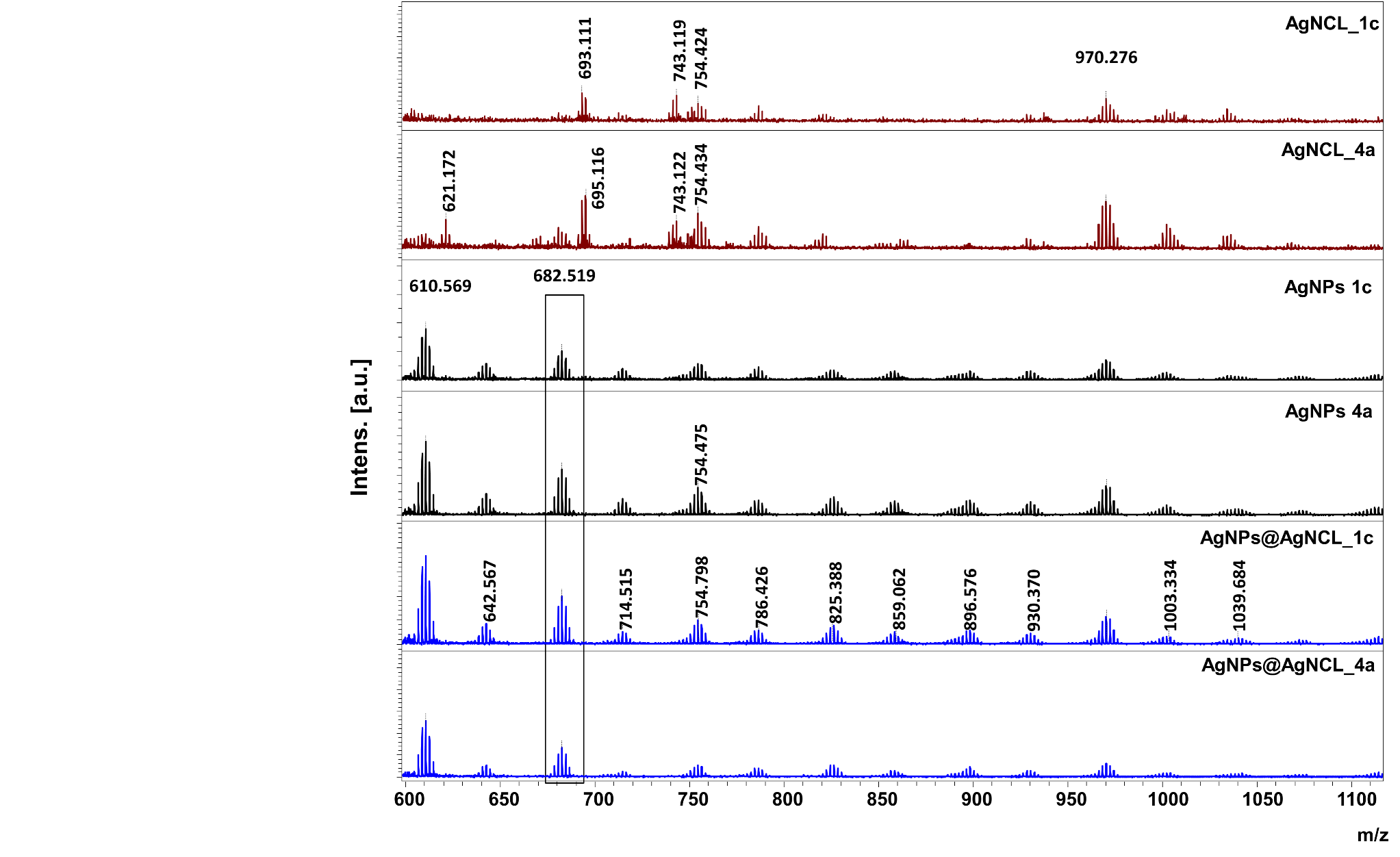


Figure S1 . MS spectrum showing m/z ranged between 600 and 1100.

Supplement: Supplementary file 1 — Additional file 1: Fig. S1. MS spectrum showing m/z ranged between 600 and 1100 [file 11671_2023_3777_MOESM1_ESM.docx]
